# Spectroscopy and Excited-State Dynamics of Methyl Ferulate in Molecular Beams

**DOI:** 10.1021/acs.jpca.4c05792

**Published:** 2024-12-17

**Authors:** Ivan Romanov, Yorrick Boeije, Josene M. Toldo, Marianna T. Do Casal, Mario Barbatti, Wybren Jan Buma

**Affiliations:** † Van’t Hoff Institute for Molecular Sciences, University of Amsterdam, Science Park 904, Amsterdam 1098 XH, the Netherlands; ‡ Department of Chemical Engineering and Biotechnology, University of Cambridge, Philippa Fawcett Drive, Cambridge CB3 0AS, U.K.; § Department of Physics, Cavendish Laboratory, University of Cambridge, JJ Thomson Avenue, Cambridge CB3 0HE, U.K.; ∥ Aix Marseille University, CNRS, ICR, Marseille 13397, France; ⊥ UCBL, ENS de Lyon, CNRS, LCH, UMR 5182, Lyon 69342, France; # Department of Chemistry, Quantum Chemistry and Physical Chemistry Division, KU Leuven 3001, Leuven, Belgium; ∇ Institut Universitaire de France, Paris 75231, France; ○ Institute for Molecules and Materials, FELIX Laboratory, Radboud University, Toernooiveld 7c, Nijmegen 6525 ED, the Netherlands

## Abstract

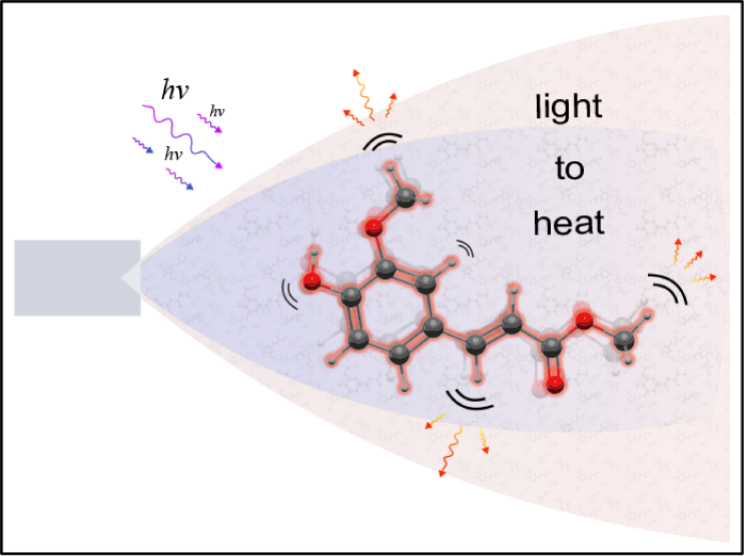

The spectroscopic and dynamic properties of methyl ferulate—a naturally occurring ultraviolet-protecting filter—and microsolvated methyl ferulate have been studied under molecular beam conditions using resonance-enhanced multiphoton ionization spectroscopy in combination with quantum chemical calculations. We demonstrate and rationalize how the phenyl substitution pattern affects the state ordering of the lower excited singlet state manifold and what the underlying reason is for the conformation-dependent Franck-Condon (FC) activity in the UV-excitation spectra. Studies on microsolvated methyl ferulate reveal potential coordination sites and the influence of such coordination on the spectroscopic properties. Our quantum chemical studies also enable us to obtain a quantitative understanding of the dominant excited-state decay routes of the photoexcited ππ* state involving a ∼3 ns intersystem crossing pathway to the triplet manifold—which is much slower than found for coumarates—and a relatively fast intersystem crossing back to the ground state (∼30 ns). We show that a common *T*_1_/*S*_0_ crossing can very well explain the observation that *T*_1_ lifetimes are quasi-independent of the phenyl substitution pattern.

## 1 Introduction

Molecules that absorb UV radiation are well known in both natural and artificial forms. Their use can broadly be divided into compounds that are merely used to absorb photons, such as those found in sunscreens and other UV filters,^1^ and compounds in which the photon energy is converted into other targeted forms of energy, such as chemical energy (photosynthesis)^2,3^ or mechanical energy (molecular motors).^4,5^ Less widely known applications focus on converting photon energy into thermal energy. Photon-to-heat conversion may be achieved indirectly through the photochemical formation of strained products that release heat by a catalytic back-reaction.^6^ Alternatively, direct photon-to-heat conversion from electronically excited states has started to attract considerable attention because of the potential of such so-called molecular heaters to boost crop growth, thereby addressing the increasing societal problem of food security.^7^

Cinnamates are a class of compounds widely used in nature as UV screening compounds.^1^ As such, they represent a natural starting point for further development of sunscreen components and molecular heaters. By now, a wide range of chemically modified cinnamates have been explored for their properties.^8−15^ Such properties ideally involve a large UV absorption coefficient and fast internal conversion to the ground state without any long-lived electronically excited states. The latter requirement implies facile access to a real crossing between the potential energy surfaces of the electronically excited and the ground state, i.e., a conical intersection.^16−18^ For a long time, coumarates employed in commercial sunscreens (2-ethylhexyl-4-methoxycinnamate (EHMC)) were assumed to fulfill such conditions. However, Tan et al. showed in 2014 that their excited-state dynamics also involve a long-lived electronically excited state^19^ that was subsequently identified by Ebata et al. as being the lowest excited triplet state.^20^ Such long-lived states are clearly detrimental to the efficacy of a sunscreen. As such, the study by Tan et al. prompted a renewed interest in the photochemical characterization of natural and artificial UV filters.^1,7,18,21−31^

Optimization—and ultimately the rational design—of novel cinnamate-based compounds requires detailed insight into their spectroscopic properties and excited-state dynamics, and in particular, how these are modified by substitutions.^32^ Initial studies revealed that these properties are delicately determined by the three lower-lying excited singlet states, the V(ππ*), V′(ππ*), and ^1^nπ* states, with the V(ππ*) state being the strongly allowed HOMO → LUMO excitation and the V′(ππ*) state having a large HOMO → LUMO+1 character.^33^ In coumarates, the V(ππ*) and V′(ππ*) states are nearly degenerate, with the V′(ππ*) state being the lowest one for vertical excitation. Adiabatically, however, the ^1^nπ* state becomes the lowest electronically excited singlet state.^34,35^ This has important consequences as this state enables efficient intersystem crossing (ISC) to the triplet manifold.^36,37^ On the other hand, in sinapates, which feature two meta-substituted electron-donating methoxy-groups, this order is completely reversed with the strongly allowed V(ππ*) state being the lowest excited state for both vertical as well as adiabatic excitation.^12,29^ Interestingly, the assignment to the V(ππ*) state was originally made by comparing experimentally observed and computationally predicted Franck–Condon (FC) activity in the excitation spectrum.^12^ Follow-up studies showed, however, that the structural relaxation in the excited state is highly conformer dependent.^38^ Despite the energetic inaccessibility of the ^1^nπ* state in sinapates, it was found that decay of the V(ππ*) state may still proceed via ISC, although to a much lesser extent than in coumarates.^9,38^

**Scheme 1:**
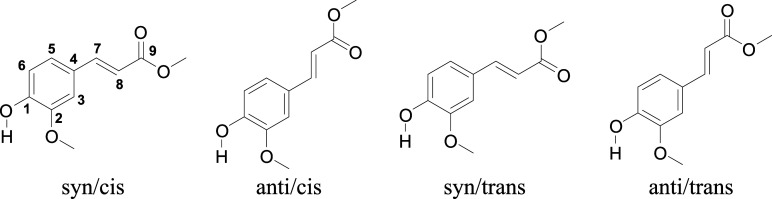
Four Lowest-Energy Stable Conformers of Methyl Ferulate with Atom Labeling as Used throughout the Text *syn/anti* indicates the relative orientation of the O–H and C_7_=C_8_ bonds, while *cis/trans* refers to the relative orientation of the C_7_=C_8_ and C_9_=O bonds.

Methyl ferulate (MF, Scheme 1), which can be seen as coumarate with a single meta-substituted methoxy group instead of the two that are present in sinapates, is an ideal candidate to further our understanding of the influence of meta-substituted methoxy groups on the spectroscopic and dynamic properties of the lower electronically excited singlet states. A recent report on ferulic acid (FA) suggests that the triplet manifold is efficiently populated similar to coumarates, even though the ^1^nπ* excitation energy was determined to be higher than those of the V(ππ*) and V′(ππ*) states.^9^ Here, we present detailed experimental and quantum chemical studies of the UV excitation spectrum of MF and show how they are influenced by solvation. These studies are complemented by IR-depletion spectra for the further assignment of the specific conformation. We show that the vibrational activity in the excitation spectra of these conformers is delicately dependent on the molecular conformation. One should thus be cautious with the assignment of the character of the electronically excited state on the basis of observed vibronic activity.

We also investigate in detail the decay pathways available to photoexcited MF, aiming to obtain not only a qualitative understanding of these pathways but also a quantitative agreement between experiment and theory. These studies provide insight into the ISC pathways of substituted cinnamates. Importantly, they also reproduce at a quantitative level experimental observations and thereby provide a consistent explanation for the observation that the decay rate of the lowest excited triplet state to the ground state is quasi-independent of the substitution pattern. All in all, these studies pave the way for further rational optimization of cinnamates for applications as UV absorbers and in photothermal materials.

## 2 Methods

### 2.1 Experimental Section

Methyl ferulate was used as purchased from Sigma-Aldrich. Resonance-Enhanced Two Photon Ionization (R2PI), UV–UV depletion, IR–UV depletion, and pump–probe studies have been performed on a molecular beam setup described in detail before.^39^ In these experiments, methyl ferulate was heated to 140 °C in an *in situ* glass container just before the pulsed valve, and its vapor was seeded into a supersonic expansion of 2.0 bar neon using a pulsed valve (General Valve Iota One) with an orifice diameter of 0.5 mm that was kept 5 °C higher in temperature to avoid clogging. Typically, a pulse duration of 180–220 μs was used. Because of the unavoidable presence of water vapor in gas tubes and the sample container itself, this expansion led to not only supersonically cooled methyl ferulate but also clusters of methyl ferulate with water. The molecular beam thus created was subsequently skimmed with a 2.5 mm skimmer and directed into the ionization region, where mass-resolved ion detection was performed using a reflectron time-of-flight spectrometer (R.M. Jordan Co.).

One- and two-color R2PI experiments have been performed using a frequency-doubled Sirah Cobra-Stretch dye laser operating on DCM and pumped by a Spectra Physics Lab-190 Nd:YAG laser. In the two-color R2PI and pump–probe experiments, ionization was performed with a Neweks PSX-501 ArF excimer laser (193 nm, 6.42 eV). To obtain nonsaturated (1 + 1’) excitation spectra, typical excitation energies of <10 μJ needed to be used, while typically ionization pulse energies of 0.05 mJ were employed to keep the one-color signal from the ionization laser as small as possible. Experiments focusing on low-intensity transitions in the excitation spectrum were performed in a one-color (1 + 1) scheme with typical pulse energies of 0.3 mJ. In pump–probe studies of the excited-state dynamics, the ion yield at the molecular mass was detected in analog mode and monitored as a function of the delay between the excitation and ionization laser. This delay was scanned by using a Stanford Research Systems DG645 delay generator.

UV–UV depletion experiments were performed by depopulating the ground state with a frequency-doubled Sirah Precision Scan dye laser operating on DCM and pumped by a Spectra Physics Lab-190 Nd:YAG laser. These experiments typically used pulse energies of 1.5–2 mJ for the depletion step and a time delay between the depletion laser and the excitation–ionization probe lasers of 150 ns. For the IR–UV depletion experiments, an IR pump beam in the range of 2870–3440 cm^–1^, with a typical pulse energy of 1 mJ, was produced by difference frequency, which involved mixing the output of the Sirah Precision Scan dye laser, operating on LDS 789 (779–815 nm), with the fundamental output of a Nd:YAG laser in a LiNbO_3_ crystal. This IR beam was partially focused by a lens with a focal length of 30 cm, placed 20 cm from the intersection with the molecular beam. The excitation–ionization probe lasers were not further focused but were reduced to an appropriate size using pinholes. In these experiments, a typical time delay of 200 ns between the depletion and probe lasers was employed.

### 2.2 Computational Section.

Ground state geometries of MF and MF-H_2_O conformers were optimized with Density Functional Theory (DFT) at the ωB97XD/cc-pVDZ level,^39,40^ while Time-Dependent (TD)-DFT at the same level was employed to optimize geometries in electronically excited states. These geometries were subsequently used to determine vertical and adiabatic excitation energies for electronically excited singlet and triplet states using TD-DFT as well as the combined Density Functional Theory and MultiReference Configuration Interaction (DFT/MRCI)^40,41^ method. TD-DFT excitation energies were computed at the ωB97XD/aug-cc-pVTZ level, while DFT/MRCI energies were obtained from DFT calculations at the BH-LYP/def2-TZVP level in combination with MRCI calculations. For the latter calculations, a modified set of the original empirical parameters^40^ was used (*p*_1_ = 0.629, *p*_2_ = 0.611, *p*_J_ = 0.119, p[0] = 8.000, α = 0.503) as suggested in Abiola et al.^7^ In these calculations, the cutoff energy for the configuration selection was set to 1 Hartree to ensure that all relevant electronic configurations would be included in the calculations.

Simulations of vibrationally resolved excitation spectra were performed by ωB97XD/cc-pVDZ (TD)DFT calculations of the force fields in ground and electronically excited states followed by calculations of the pertinent Franck–Condon factors. For comparison with the experimental spectra, theoretically calculated vibrational frequencies were scaled with a uniform scaling factor of 0.953.^42^ DFT and TDDFT calculations were performed with the Gaussian 16, Rev. A.03 suite of programs^43^ except for the DFT/MRCI calculations for which the DFT part was calculated with the Turbomole 7.5.0 package.^44^ The MRCI part was calculated using the parallelized version of the MRCI code.^45^

For the singlet states, scans of the potential energy curves were obtained by using linearly interpolated intrinsic coordinates (LIIC). In these scans, the energy of electronic states was calculated at molecular geometries that for the first 20 points were linearly interpolated between the equilibrium structure of the V(ππ*) state of the *E* isomer and the geometry at which there is a minimum energy surface crossing between the S_1_ and S_0_ states. For the subsequent 20 points, the geometries were linearly interpolated between the latter geometry and the V(ππ*) state of the *Z* isomer. For the *T*_1_ state, on the other hand, a relaxed surface scan was obtained in which interpolated geometries were optimized using unrestricted DFT (uDFT) while constraining the C_4_—C_7_=C_8_—C_9_ dihedral angle. Corresponding single-point singlet energies were computed at each geometry with ωB97XD/aug-cc-pVTZ TDDFT calculations. Minimum energy surface crossings (MESX) between singlet states and between singlet and triplet states were computed using an in-house modified version of the CIOpt program developed by Levine and Martinez,^46^ which employs a sequential penalty function to optimize state crossings without the need for nonadiabatic coupling vectors. Initial values for the penalty weight and smoothing parameters were set to 3.5 and 0.025 Hartree, respectively.

Spin-orbit coupling matrix elements (SOCMEs) were evaluated at several optimized structures, including the optimized triplet structures and *T*_1_/*S*_0_ MESX. Before the SOC calculation, TDDFT single-point energies were computed at the ωB97XD/aug-cc-pVTZ level with 6 d and 10 f basis functions and symmetry turned off. SOCME values were then obtained using the Breit–Pauli spin-orbit Hamiltonian with the effective charge approximation,^47^ as implemented in the PySOC program.^48^

## 3 Results and Discussion

### 3.1 Spectroscopy Methyl Ferulate

**Figure 1:**
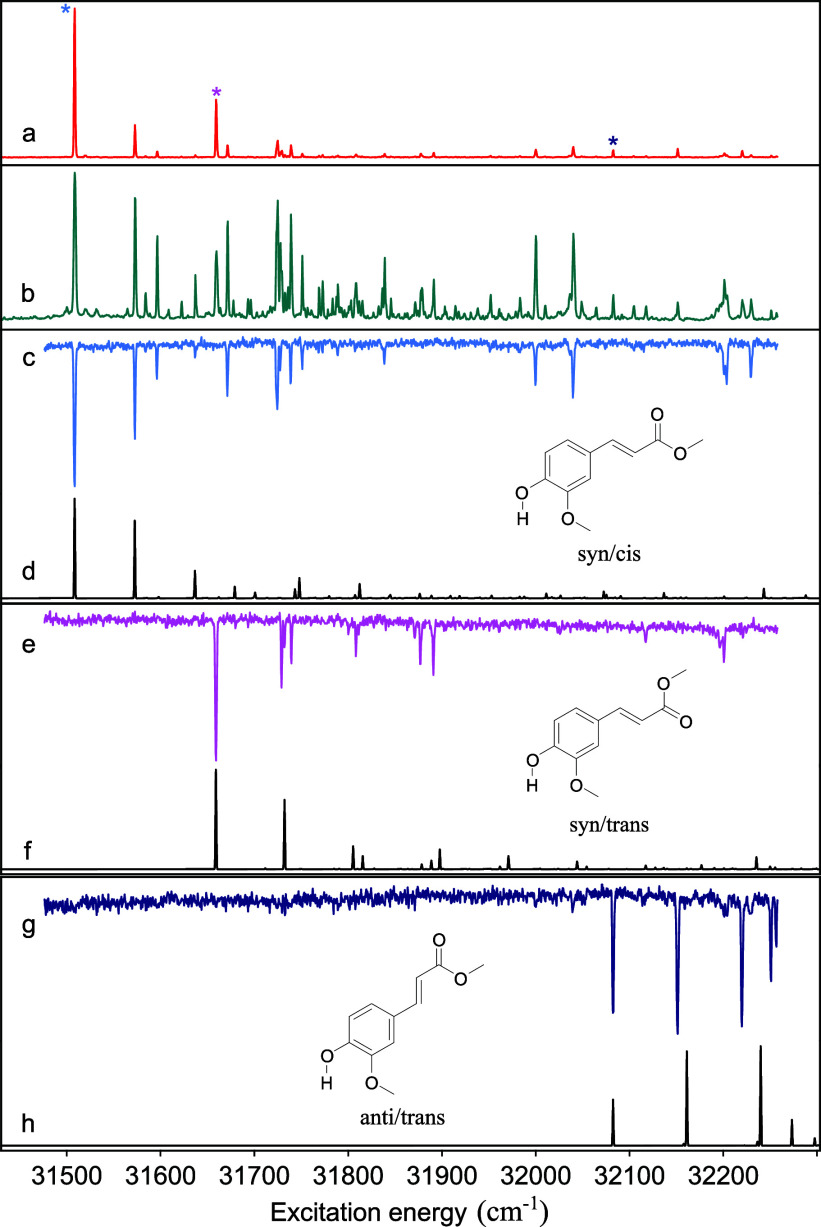
(1 + 1’) R2PI (a) and (1 + 1) R2PI (b) excitation spectra of MF. UV–UV depletion spectra of three conformers depleted at 31508.5 cm^–1^ (c), 31659.6 cm^–1^ (e), and 32083.7 cm^–1^ (g)—indicated by asterisks in (a)—which are assigned to the *syn/cis*, *syn/trans*, and *anti/trans* conformations, respectively, based on the TD-DFT-computed FC spectra depicted in (d), (f), and (h).

Figure 1 displays the R2PI excitation spectrum of MF under nonsaturated (a) and saturated (b) excitation conditions. The former excitation conditions have been employed to determine accurately the Franck–Condon (FC) factors for active vibrations and compare them with quantum chemical predictions. The latter, on the other hand, serve as a reference for comparing experimentally observed vibrational frequencies in the electronically excited state with theoretically predicted ones. As expected, these spectra are quite similar to the spectrum reported previously for ethyl ferulate (EF)^30^ albeit that the EF spectrum—as also indicated in the previous study—was strongly saturated. In the study reported in ref. (30) two EF conformers were observed. Interestingly, UV–UV depletion spectroscopy on MF (Figure 1c,e, g) allows us to identify three species contributing to these spectra with 0–0 transitions at 31508.5, 31659.6, and 32083.7 cm^–1^, each displaying a dominant vibrational activity of a low-frequency bending mode similar to that observed for EF.^30^ The lowest-energy origin transition (31508.5 cm^–1^) is close to the two origin transitions identified for EF (31491.1 and 31507.0 cm^–1^).^30^ Previous studies on the bare chromophore FA identified two conformers with origin transitions at 31 780 and 32 095 cm^–1^.^9^ Comparison with the *syn/cis* conformer of MF, which has the lowest excitation energy, thus leads to the conclusion that methylation of the carboxyl group leads to a red-shift of 271 cm^–1^. It is interesting to notice that this is opposite to what is observed for coumaric acid^49^ and sinapic acid^10^ for which methylation leads to blue-shifts of 116 and 304 cm^–1^, respectively.^19,29^

In the case of sinapic acid, one of the arguments to assign the absorbing state as the V(ππ*) state and not the V′(ππ*) state was the observed Franck–Condon activity.^12^ For the V(ππ*) state, quantum chemical calculations predicted an extensive C_4_—C_7_=C_8_ bend progression, while for the V′(ππ*) state, such activity was predicted to be much smaller. The present study on MF shows, however, that such an argument should be used with caution as the depletion spectra of the two lowest-energy conformers (c and e) display limited C_4_—C_7_=C_8_ bend activity leading to a “stairs” pattern (intensity distribution over the Franck–Condon progression with the 0–0 transition having the highest intensity), while for the highest-energy conformer (g), a “quartet” pattern (intensity distribution over the Franck–Condon progression with the 0–1 and 0–2 transitions having a higher intensity than the 0–0 and 0–3 transitions) with extensive activity of this mode is observed. We thus conclude that the Franck–Condon activity of the C_4_—C_7_=C_8_ in-plane bending mode depends not only on the nature of the excited state but also on the specific conformer.

Quantum chemical calculations enable us to obtain more insight into these observations and understand how the substitution pattern on the phenyl group affects the spectroscopic properties of these compounds. These calculations find four stable MF ground-state conformers (Scheme 1) with relative energies given in Table 1, noticing that the differences between the DFT and DFT/MRCI results are minor. Similar to previous conclusions on the stability of conformers of ethyl ferulate,^30^ methyl sinapate,^29^ ferulic acid,^9^ and sinapic acid,^10^ we find that the *syn/cis* conformer is the most stable one and will thus have the largest contribution to the R2PI spectrum in Figure 1a, given that the predicted V(ππ*) oscillator strengths of all conformers are approximately equal. For this reason, we focus first on the excited state properties of the *syn/cis* conformer before returning to the assignment of the individual conformers.

**Table 1:** Relative Energies of MF Conformers Calculated at the ωB97XD/aug-cc-pVTZ Level and Associated Room Temperature (RT) Predicted Population Ratios

Conformer	(kcal/mol)	RT Population^a^	RT Population (Experimental)^b^
*syn/ci*s	0	1	1
*anti/cis*	0.28	0.63	-
*syn/trans*	0.67	0.32	0.35
*anti/trans*	1.28	0.12	0.13

a Relative populations inclusive of enthalpic contributions.

b Determined from integrated intensities of bending vibration progression bands.

According to our DFT/MRCI calculations, the manifold of the lower electronically excited singlet states of the *syn/cis* conformer of MF is composed of three states, which are ordered according to their vertical excitation energies as V(ππ*) < V′(ππ*) < ^1^nπ* (Table 2). Such an ordering—although with slightly higher excitation energies—is also found by TDDFT calculations on MF (Table S1), as well as by TDDFT calculations on FA.^9^ CASPT2 calculations on EF predict that for vertical excitation, the ^1^nπ* state is *S*_2_.^30^ Further comparative studies on EF and MF would thus be of interest but are outside the scope of the present work.

**Table 2:** Vertical and Adiabatic DFT/MRCI Excitation Energies (eV) of the Lower-Lying Electronically Excited Singlet States of the *syn/cis* Conformer of MF with Oscillator Strength of the Corresponding Transition Given in Parentheses

Transition	Vertical	Adiabatic
V(ππ*)	4.02 (0.56)	3.81^a^ (0.67)
V’(ππ*)	4.40 (0.25)	4.22 (0.46)
^1^nπ*	4.45 (4 × 10^–4^)	3.83 (6 × 10^–5^)

a Experimental adiabatic excitation energy is 3.91 eV.

**Figure 2:**
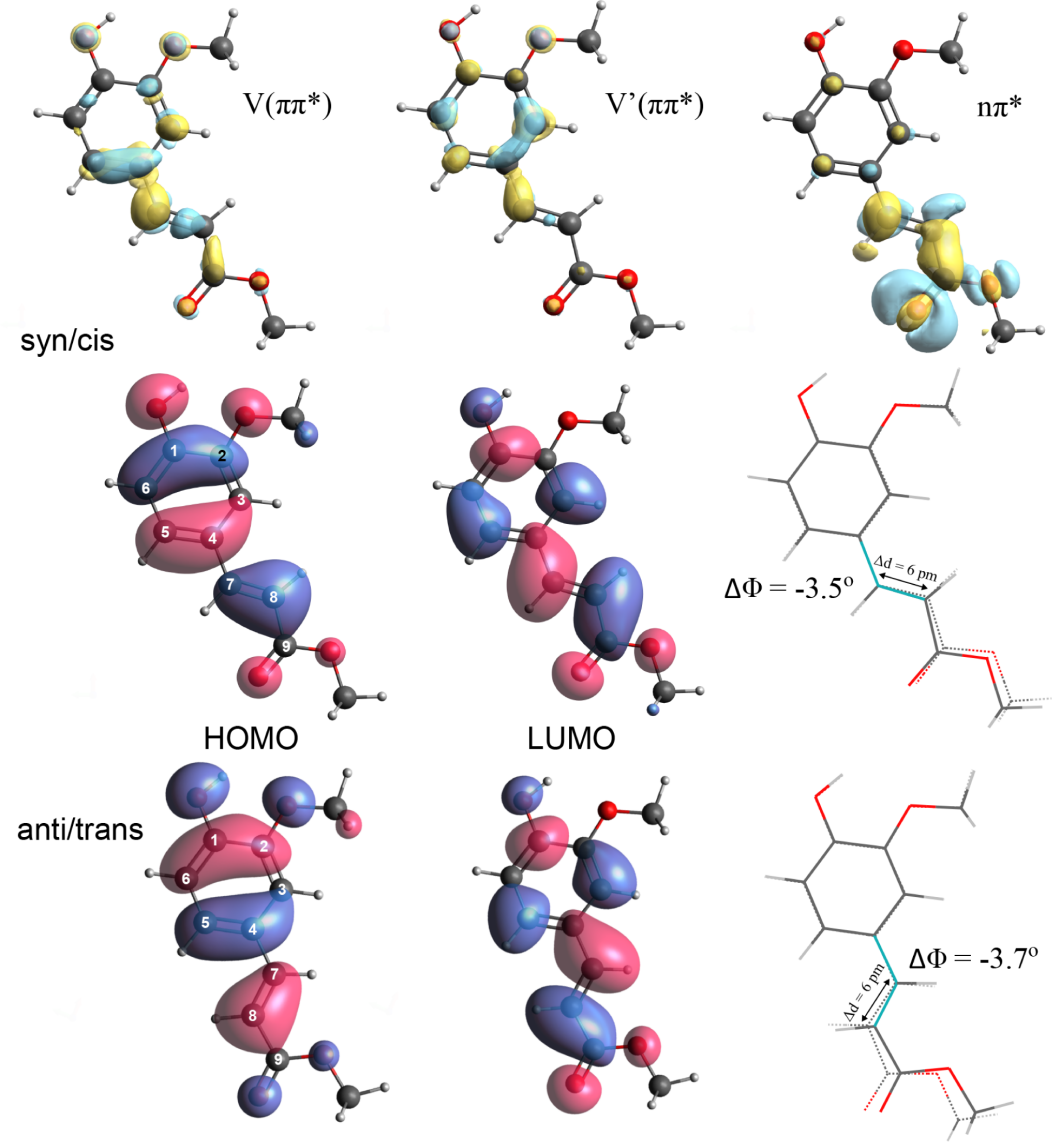
Top: Electron density difference (EDD) maps for the V(ππ*), V′(ππ*), and ^1^nπ* states of the *syn/cis* conformer of MF, with blue and yellow indicating hole and electron density, respectively. Middle and bottom panels show Kohn–Sham HOMO and LUMO orbitals for *syn/cis* and *anti/trans* conformers, respectively. The right structures indicate the change of the C_4_—C_7_=C_8_ angle Δ in the V(ππ*) state of the *syn/cis* (middle) and *anti/trans* (bottom) conformers compared to the ground state.

The V(ππ*) state is described by a pure HOMO → LUMO transition (Figure 2 and Table S2) and has a much larger oscillator strength than the configurationally mixed V′(ππ*) and ^1^nπ* states. Inspection of the molecular orbitals rationalizes why the V(ππ*) state is red-shifted with respect to MC, as the methoxy group in the ortho-position raises the energy of the HOMO but does not affect the LUMO due to the nonbonding character at the ortho-position. The large oscillator strength of the *S*_0_ → V(ππ*) transition arises from the spatial overlap between the HOMO and LUMO in combination with a relatively small charge transfer from the phenyl ring to the carbonyl tail accompanying the transition (see electronic difference density (EDD) maps in Figure 2). These EDD maps show that excitation of the V(ππ*) state also leads to transfer of electron density from the C_7_=C_8_ to the C_4_–C_7_ regions, which thereby acquire less and more double-bond character, respectively. As a result, the C_4_–C_7_=C_8_ angle is changed upon excitation, leading to the observed activity of the associated in-plane bend vibration. Similar to the V(ππ*) state, π-electronic density is transferred upon excitation of the V′(ππ*) state from the phenyl moiety to the C_4_–C_7_ region. It is noteworthy that the EDD maps of the V(ππ*) and the V′(ππ*) states also show significant differences in the phenyl ring arising from the differences in electron distribution in the HOMO and HOMO–1 orbitals. Finally, the EDD map for excitation of the nπ* state shows how electron density from the oxygen lone pair is displaced to the π-plane, in particular, the acrylate region.

Geometry relaxation of the molecule in these three electronically excited states lowers the energy of the ^1^nπ* state significantly. Nevertheless, its adiabatic excitation energy is still higher than that of the bright V(ππ*) state (Table 2). As a result, the three states adiabatically become ordered as V(ππ*) < ^1^nπ* < V′(ππ*). Similar to MS, we thus find that the V(ππ*) state of *syn/cis* MF is both vertically as well as adiabatically S_1_, as opposed to MC, where the ^1^nπ* state is adiabatically the lowest excited singlet state.^19^ Both the predicted excited state ordering as well as the large oscillator strength of the V(ππ*) state lead us to the conclusion that the excitation spectrum observed in Figure 1 is associated with the V(ππ*) state.

Our DFT/MRCI calculations predict that for the *syn/trans* and *anti/trans* conformers, the ^1^nπ* state is adiabatically slightly lower in energy than the V(ππ*) state (Table S3), which is in contrast with the TD-DFT calculations that predict that adiabatically the ^1^nπ* state is at a significantly higher energy (Table S1). We contend, however, that for the present experiments, it is not of direct importance whether the ^1^nπ* state is adiabatically above or below the V(ππ*) state. In our experiments, we excite adiabatically a strongly allowed electronically excited state, which thus must be a ππ* state. Internal conversion to the ^1^nπ* state is expected to be slow as the energy barrier that must be overcome will be quite high because of the large differences in equilibrium geometry of the two states. Previous calculations find indeed that the CI between the V(ππ*) and ^1^nπ* states, which would accelerate such an internal conversion process, is more than 0.5 eV above the minimum of the V(ππ*) state.^30^ We thus conclude that for all conformers, the excitation spectrum observed in Figure 1 is associated with the V(ππ*) state.

Such an assignment is further supported by the calculated vibrationally resolved excitation spectra (Figure 1d,f,h), which for the V(ππ*) state show a similar vibronic activity as observed experimentally. What is important to notice is that the observed “stairs” Franck–Condon patterns of the lowest-energy conformers (Figure 1c,e) are reproduced computationally for the V(ππ*) excitation spectra of the *syn/cis* and *syn/trans* conformers, as well as the “quartet” pattern found in the V(ππ*) excitation spectra of the *anti/cis* and *anti/trans* conformers (see Figure S1 for the predicted spectrum of *anti/cis*). Moreover, for the latter two conformers, the Franck–Condon patterns calculated for excitation of the V′(ππ*) state are in stark contrast with what is observed experimentally (Figure S2), thereby excluding the possibility that for these conformers, the V′(ππ*) state is adiabatically the lowest excited singlet state.

The above considerations, in combination with the calculated population ratios, lead to the conclusion that the depletion spectra in Figures 1c and 2e should be assigned to the *syn/cis* and *syn/trans* conformers, respectively. Such a conclusion is in agreement with the lower adiabatic excitation energy predicted for the former. Based on the “quartet” pattern observed in the depletion spectrum of Figure 1g, the same considerations lead one, however, also to conclude that this spectrum should be assigned to one of the *anti* conformers. The low intensity of the 32083.7 cm^–1^ band and the experimentally measured C_4_—C_7_=C_8_ bend frequency (69 cm^–1^)—which is closer to the frequency predicted for the *anti/trans* conformer (73 cm^–1^) than that for the *anti/cis* conformer (62 cm^–1^)—would tend to favor an assignment to the *anti/trans* conformer. Such an assignment implies, however, also that the *anti/cis* conformer, which is predicted to be the one but most stable conformer, is absent in our experiments. Rotationally resolved excitation spectra or ground-state rotational spectroscopy could, in this respect, allow to come to a conclusive assignment.

Given the conclusion that the *syn* and *anti* conformations display distinct Franck–Condon activity of the C_4_—C_7_=C_8_ bending mode, while previously it was assumed that these differences indicate excitation of different electronic states, it is of interest to further understand the underlying reasons for these distinct Franck–Condon activities. The excited-state displacements associated with these activities can qualitatively be understood by inspection of HOMO and LUMO (Figure 2). These show for the HOMO of the *syn/cis* conformer an antibonding interaction between the π orbitals of C_3_ and C_7/8_ while for the LUMO, part of this antibonding interaction is replaced by a bonding interaction. A HOMO → LUMO excitation will thus favor a reduction of the C_4_—C_7_=C_8_ angle or, in other words, induce Franck–Condon activity in the pertaining normal mode. For the HOMO of the *anti* conformers, on the other hand, the antibonding interaction between the π orbitals of C_5_ and C_7/8_ is larger than the antibonding interaction between the π orbitals of C_3_ and C_7/8_ in the *syn* conformers (Figure 2). As a result, an increased Franck-Condon activity is to be expected in the C_4_—C_7_=C_8_ bending mode, as is indeed observed experimentally. A similar conclusion is drawn when the steric interactions between H_8_ and H_3_ and between H_7_ and H_4_ in the *syn* conformers are compared with the analogous interactions between H_7_ and H_5_ and between H_4_ and H_8_ in the *anti* conformers since the latter distances are smaller than the former. Both aspects (interactions between π orbitals and steric interactions between H atoms) thus favor a larger activity of the C_4_—C_7_=C_8_ bending mode in the excitation spectrum of the *anti* conformers.

The above observations indicate that one should be cautious when assigning the electronic nature of the excited state based on the Franck-Condon activity of a single conformer, in particular, when there is an asymmetric substitution in the phenyl ring. In contrast, in MC and MS, where the phenyl ring is symmetrically substituted, it might very well be that the *anti/syn* Franck–Condon distinction is less pronounced.^12^ In this respect, it is interesting to notice that for MS, it has been concluded that excitation of the V(ππ*) state is accompanied by a larger change in the C_4_—C_7_=C_8_ angle than in MF and that this change is larger for the *syn/cis* conformer than for the *anti/cis* conformer.^38^

### 3.2 Influence of Solvation on the Spectroscopy of Methyl Ferulate

**Figure 3:**
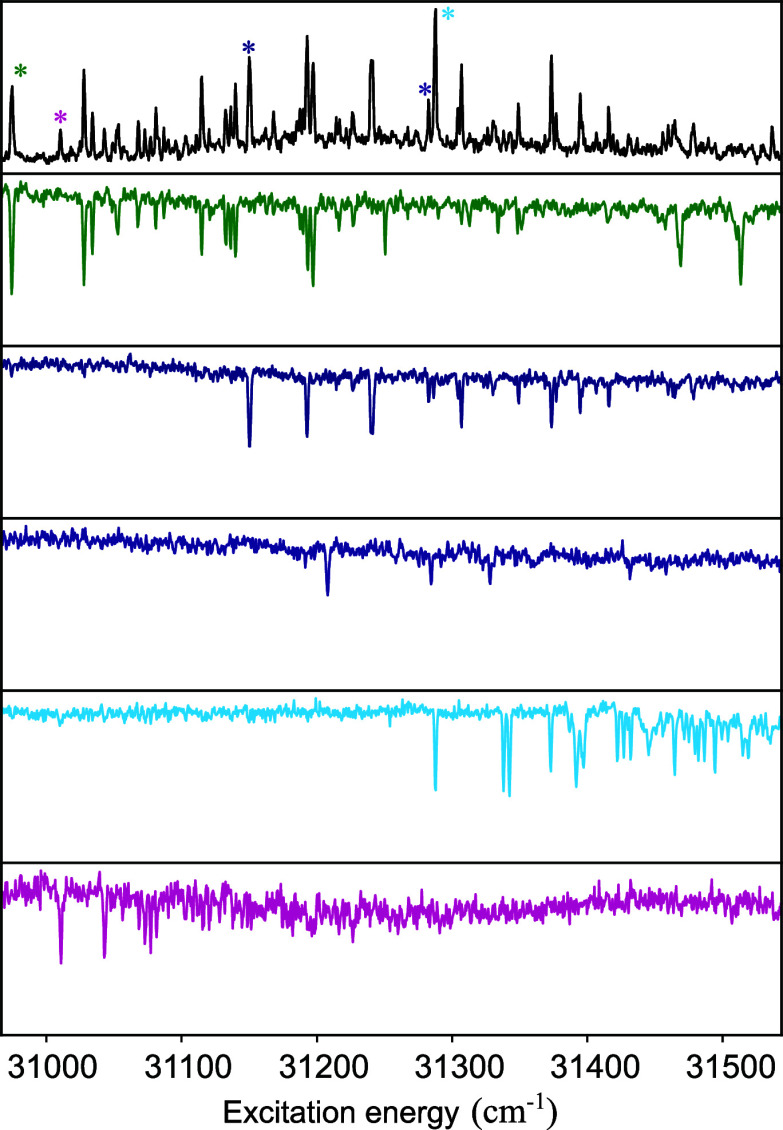
(1 + 1’) R2PI spectrum (black) and UV–UV depletion spectra of MF-H_2_O depleted at excitation energies of 30 978 cm^–1^ (green), 31 153 cm^–1^ (dark blue), 31 211 cm^–1^ (purple), 31 290 cm^–1^ (light blue), and 31 014 cm^–1^ (pink) indicated with corresponding stars in the R2PI spectrum.

The R2PI spectrum of microsolvated MF obtained at the mass of the MF-H_2_O molecular ion is shown in Figure 3. The lowest-energy band in this spectrum is observed at 30977.5 cm^–1^, implying that the coordination of a water molecule red-shifts the lowest excitation energy observed for MF (Figure 1) by 531 cm^–1^, which is less than observed in MS (822 cm^–1^)^29^ and MC (641 cm^–1^).^34,35,50^ UV–UV depletion spectroscopy enables the identification of five MF-H_2_O structures (Figure 3). These spectra inevitably show vibronic progressions more complicated than those of uncoordinated MF because of the additional intermolecular MF-H_2_O vibrational modes.

**Figure 4:**
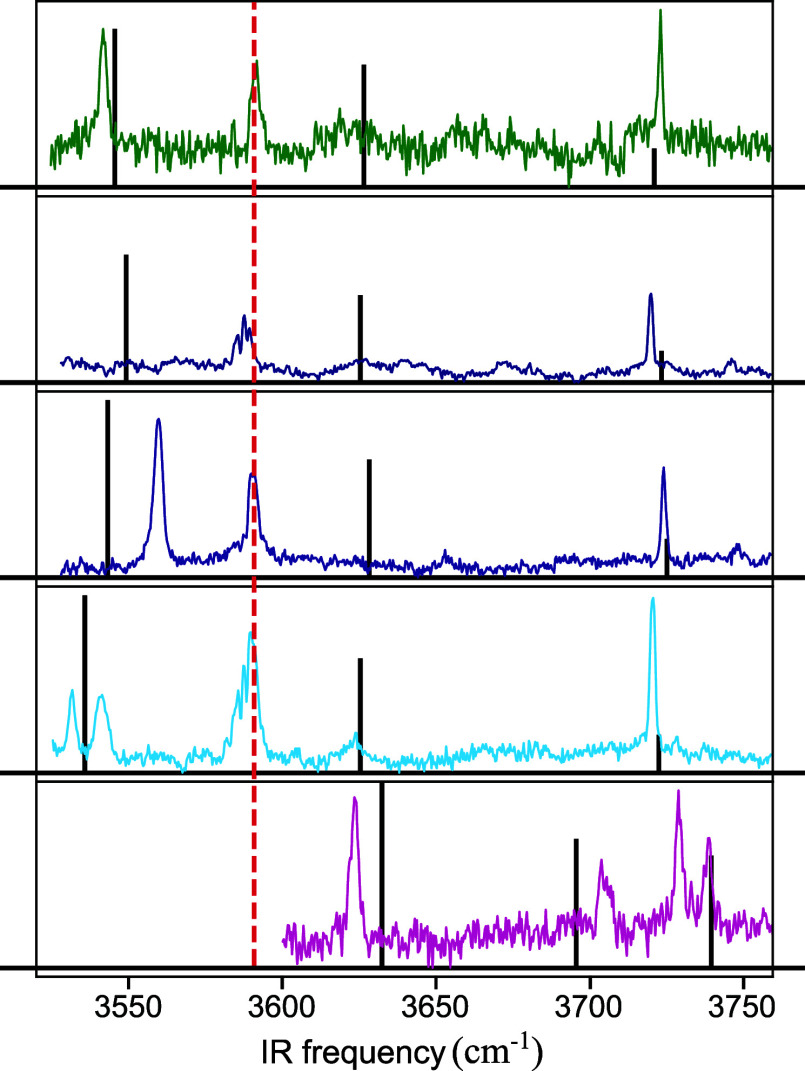
IR–UV depletion spectra of the MF-H_2_O conformers. The colored spectra match the conformers found in the UV–UV depletion spectra in Figure 3. For the pink conformer, the region below 3600 cm^–1^ is not depicted since scans with less averages did not show any bands in this region. Red dashed lines indicate the frequency measured for the Ph-OH stretch in bare MF conformers (Figure S3). Calculated (normalized) IR spectra for the ground state of the assigned water cluster conformers are represented by black-stick spectra (Table 3).

In order to assign these spectra to specific MF-H_2_O structures, IR–UV depletion spectroscopy has been applied (Figure 4). IR–UV depletion spectra obtained for conformers of the bare MF molecule (Figure S3) serve in this respect as a starting point for further comparisons. These spectra show for the *syn/cis* and *anti/trans* conformers a strong OH stretch absorption band at the same frequency (3591.8 and 3591.6 cm^–1^, respectively) and at a slightly lower frequency (3590.6 cm^–1^) for the *syn/trans* conformer. For MF-H_2_O, we expect two additional OH bands. The light blue and pink spectra in Figure 4 show, however, more than three bands. We attribute these additional bands to either combination bands or excitation of more than one conformer at the employed UV probe wavelength.

**Scheme 2:**
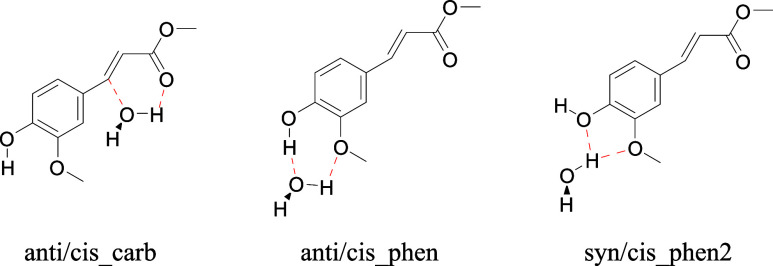
A Selection of Possible Relevant MF-H_2_O Structures of Microsolvated MF Clusters Microsolvated MF-H_2_O structures that differ in water coordination site are formally structural isomers. For the sake of simplicity, we will, however, refer to such isomers as conformers. Relative energies are given in Table S4. Possible hydrogen-bond interactions are indicated with red dashed lines.

The IR spectra of the microsolvated conformers reveal two new OH stretching mode bands: one at a much higher frequency (ν_3_) than the OH stretching frequency of bare MF (ν_2_) and one at a much lower frequency (ν_1_) (see Table 3). For four of these conformers (green, dark blue, purple, and light blue traces in Figure 4), a band is observed close to the OH stretch band of the bare molecule, implying that H_2_O coordination only mildly perturbs the MF OH stretching mode. We therefore assign these conformers to clusters designated as *carb*-coordinated water clusters (Scheme 2) in which a water molecule is coordinated to the carbonyl group, similar to how water is coordinated in MS-H_2_O clusters.^38^ The pink trace, on the contrary, does not show an IR band close to the bare MF OH stretch frequency, indicating that coordination takes place with the phenolic OH. Such a coordination disrupts the intramolecular hydrogen bond between the OH and the OCH_3_ groups in the bare molecule and replaces it with a weaker hydrogen bond of the phenolic OH with water. As a result, the Ph-OH stretch is blue-shifted by ∼112 cm^–1^.

**Table 3:** DFT Calculated and Measured the IR–UV OH Stretch Frequencies (cm^–1^) for *carb* Water Clusters and *phen2* Water Clusters (Scheme 2)^a^^b^^c^^d^

*carb* cluster	ν_1_	ν_2_	ν_3_	Color			
*anti/cis*	3545.6	3626.8 (+34.9)	3721.4	Green	3541.8	3591.9 (−)	3723.5
*syn/cis*	3549.3	3625.6 (+33.7)	3723.8	Dark blue	-	3587.8 (−4.0)	3720.4
*anti/trans*	3543.3	3628.6 (+36.7)	3725.6	Purple	3559.9	3590.7 (−0.9)	3724.5
*syn/trans*	3535.8	3625.7 (+33.8)	3722.9	Light blue	3531.8^e^	3589.8 (−0.8)	3721.0
*phen2* cluster							
*anti/cis*	3633.1	3695.1 (+103)	3740.2	Pink^f^	3623.7	3704.2 (+112)	3739.3^g^
*syn/cis*	3632.7	3696.0 (+104)	3740.1				

a ν_1_ represents the hydrogen-bonded OH stretch of coordinated H_2_O, ν_2_ the Ph-OH stretch, and ν_3_ the non-hydrogen-bonded OH stretch of coordinated H_2_O.

b Values in parentheses at ν_2_ indicate the shift in wavenumbers with respect to the Ph-OH stretch of bare MF.

c ν’ values denote experimental frequencies obtained by IR–UV depletion spectroscopy.

d The “Color” entry corresponds to line colors used in Figure 4.

e Another peak appears at 3541.4 cm^–1^.

f Experimental frequencies can be assigned to either *anti/cis* or *syn/cisphen2* configuration.

g Another peak appears at 3729.3 cm^–1^.

With the exception of the carrier of the pink trace (thereafter termed the pink conformer), all conformers have the highest-frequency band (ν_1_) around 3720–3730 cm^–1^. The similar frequency of this band for the four different conformers suggests that it is associated with the non-hydrogen-bonded OH stretch of coordinated H_2_O. It is interesting to notice that for MC-H_2_O clusters—in which water is coordinated to the phenolic OH—this band is found at 3743 cm^–1^.^36^ Such a higher frequency is in line with the stronger hydrogen bond between phenolic OH and H_2_O compared to the hydrogen bond between C=O and H_2_O. As such, it provides further confirmation of the coordination site of water in these four conformers. The observation that for the pink conformer the highest-frequency band is found at 3739 cm^–1^ further corroborates our conclusion that in this conformer water is coordinated to the phenolic OH. In line with an assignment of the lowest-frequency band (ν_1_) to the hydrogen-bonded OH stretch of coordinated water, much larger variations are observed for the lowest-frequency band (ν_1_) of these four conformers. The stronger interaction of the bond with MF results in a reduction of its frequency and makes the frequency of this mode more susceptible to small variations in bonding characteristics.

Our calculations suggest that for the structure of the pink conformer (Figure 4), there are two possible ways of coordinating a water molecule to the phenolic OH, which will be designated as *phen* and *phen2* (see Scheme 2). From an energy point of view, *phen* coordination would appear to be the most logical candidate (Table S4). Such an assignment is contradicted, however, by calculated IR spectra, which predict a phenolic OH stretch significantly red-shifted from the noncomplexed molecule (∼44 cm^–1^) and a hydrogen-bonded OH stretch of water around ∼3340 cm^–1^ (Table S5). In contrast, the IR spectrum observed for the pink conformer displays a blue-shifted phenolic OH stretch and no bands at lower frequencies. IR spectra predicted for the *anti/cis_phen2* and *syn/cis_phen2* conformations, on the other hand, show blue-shifted phenolic OH stretch and water OH stretch frequencies that nicely match experimentally observed bands (Table 3). Apparently, during the molecular beam expansion, *phen2* conformers are produced that are subsequently kinetically trapped. An interesting question that remains is, however, why *phen* conformers are not observed. Although an unambiguous assignment to either of these two *phen2* conformers is not possible on the basis of the observed IR spectrum, predicted adiabatic excitation energies (Table S6) and Franck–Condon activities tend to slightly favor *syn/cis_phen2*.

Above we have concluded that the green, dark blue, purple, and light blue traces in Figure 4 are associated with *carb*-coordinated water clusters. On the basis of the calculated adiabatic excitation energies (Table S6) of each of the MF-H_2_O conformers, these traces would be assigned to the *anti/cis_carb*, *syn/cis_carb*, *anti/trans_carb*, and *syn/trans_carb* conformers, respectively. Such an assignment is supported by the calculated ground-state energies of these conformers, which would lead to population ratios (Table S4) that qualitatively follow the intensities observed in the experimental excitation spectrum (Figure 4). Although not completely unambiguous, the experimental IR spectra are also in line with this assignment: (i) the *syn/trans_carb* conformer is predicted to have the lowest ν_1_ frequency as is indeed observed in the light blue spectrum; (ii) the *syn/cis_carb* conformer is similarly predicted to have a lower ν_1_ frequency as observed in the dark blue spectrum; (iii) the experimental IR spectrum of bare *anti/cis* MF is not known. The observation that the green spectrum is the only spectrum in which the ν_2_ band is blue-shifted with respect to the Ph-OH frequencies measured for bare MF conformers thus suggests that it is associated with the *anti/cis_carb* conformer.

### 3.3 Excited-State Dynamics of Methyl Ferulate

**Figure 5:**
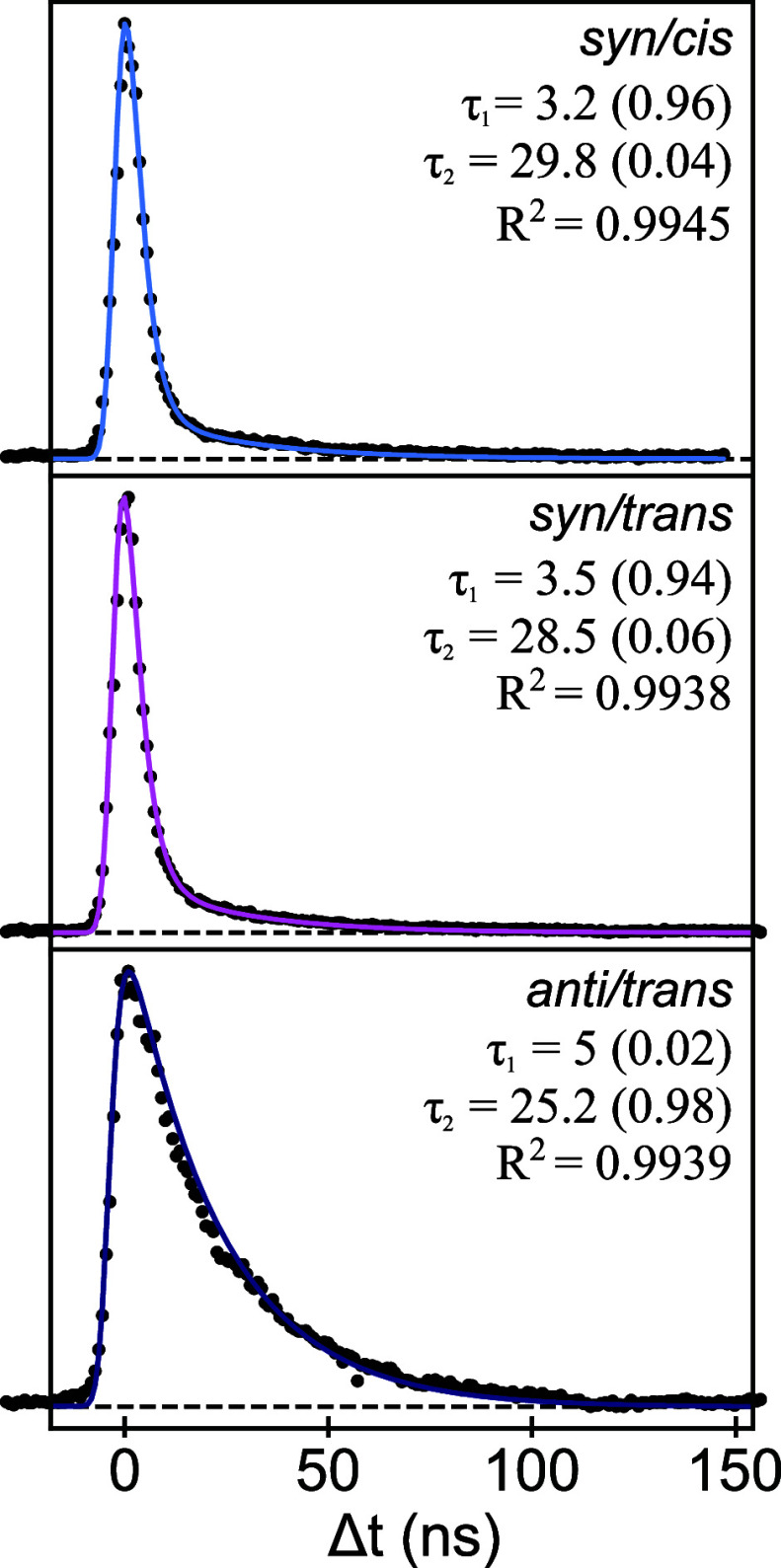
Time-resolved (1 + 1’) R2PI decay curves after excitation of the vibrationless level of the V(ππ*) state of the *syn/cis*, *syn/trans*, and *anti/trans* conformations of methyl ferulate taken with time steps of 1 ns. The solid lines are biexponential fits convoluted with a Gaussian profile, with decay times in nanoseconds, and the relative amplitude of each component given in parentheses.

After excitation of MF to the V(ππ*) state, several radiative and nonradiative decay pathways can bring the excited molecule back to the ground state. To elucidate these excited-state dynamics, pump–probe R2PI experiments have been performed. In these experiments, the vibrationless level of the V(ππ*) state of a particular conformer is excited and subsequently ionized after a chosen delay. Figure 5 displays such pump–probe traces for the three identified conformers of MF. These traces have been analyzed by taking a Gaussian profile with a width of about 6 ns as the cross-correlation of the two laser beams and convoluting this profile with a multiexponential decay. Such an analysis leads to the conclusion that the decay features a short-time (τ_1_ = 3–5 ns) component as well as a long-time (τ_2_ = 25–30 ns) component, indicating that two states are ionized within the resolution of the laser pulse, one of which is longer-lived than the other. Figure S4, which displays as an example a monoexponential fit of the decay of the *syn/cis* conformer, clearly fails to reproduce the observed decay, thereby providing further support for its biexponential nature. As we have concluded that the V(ππ*) state is adiabatically the lowest excited singlet state, we attribute the long-lived component to the decay of a triplet state. Considering that internal conversion from *T*_*n*_ to *T*_1_ is expected to take place on a ps timescale, one can safely assume that the long-lived state is the lowest excited triplet state *T*_1_. In general, one would expect a much longer lifetime for the lowest triplet state, but the short lifetimes observed here for MF nicely follow the triplet lifetimes observed in other cinnamate-based systems.^5,29,31,51^

Interestingly, all these cinnamate-based compounds—including now MF—display very similar lifetimes for the long-lived component,^8,9,19,20,29,31,34,37,50^ (20–30 ns). Moreover, for all practical purposes, this lifetime is independent of the specific conformer considered, suggesting an underlying decay mechanism that is common to all these compounds. In the following, we aim to come to a fundamental understanding of this mechanism, not only qualitatively but, in particular, also to obtain quantitative agreement between predicted and observed decay rates. To this purpose, we focus in the first instance on understanding the decay pathways of the V(ππ*) state of the *syn/cis* conformer of MF by considering (i) fluorescence, (ii) internal conversion (IC), and (iii) intersystem crossing (ISC). We estimate the radiative lifetime from Fermi’s golden rule given by:
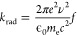
1where *e*, ϵ_0_, *m*_e_, and *c* are the elementary charge, the vacuum electric permittivity, the mass of the electron, and the speed of light, respectively. Using the DFT/MRCI-computed oscillator strength of 0.56, we obtained a radiative lifetime of 2.7 ns, which is close to the measured pump–probe lifetime of 3.2 ns. We thus conclude that radiative processes cannot be excluded from considerations on the decay paths of the V(ππ*) state.

**Figure 6:**
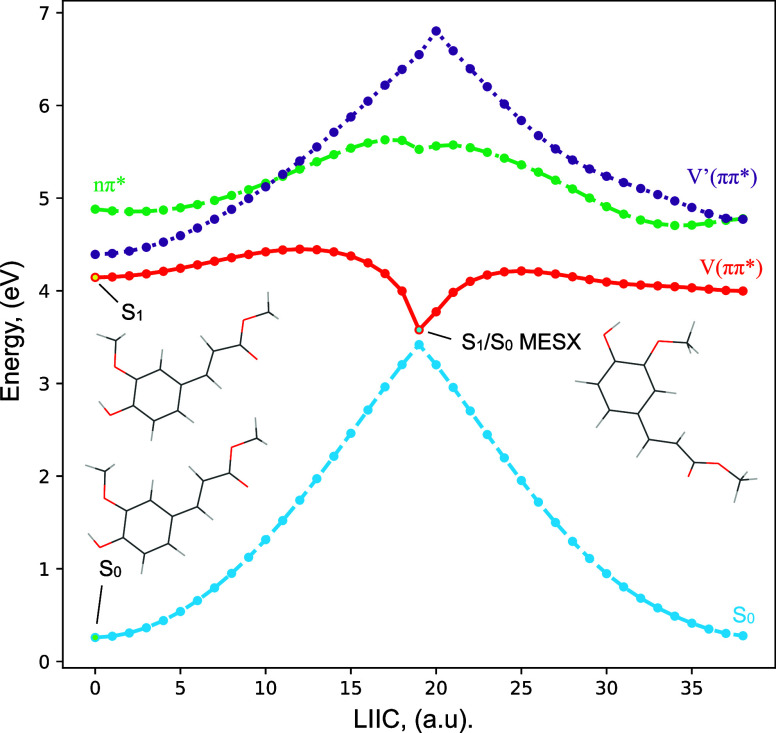
Potential energy profiles showing the evolution of ground and lower electronically excited singlet states of the *syn/cis* conformer of MF calculated at the ωB97XD/cc-pVDZ level. The curves were obtained by interpolation in internal coordinates (LIIC) between two optimized geometries. In this case, LIIC connects the equilibrium geometry of the V(ππ*) state of the C_7_=C_8_*E* isomer to the S_1_/*S*_0_ crossing, and from this last geometry to the equilibrium geometry of the V(ππ*) state of the C_7_=C_8_*Z* isomer.

One of the nonradiative processes of the V(ππ*) state that might contribute to the overall decay is internal conversion to *S*_0_. Based on the energy gap law,^52^ one expects this rate only to become appreciable in regions where the energy gap between the potential energy surfaces of two states is small. Calculations for MF show indeed a surface crossing between these two states for a twisted geometry along the C_4_—C_7_=C_8_—C_9_ dihedral angle (Figure 6). However, considering the LIIC potential energy curve connecting the  and the S_1_/*S*_0_ MESX, one finds that such a path is associated with a significant energy barrier (2455 cm^–1^), which is much larger than the available thermal energy (7 cm^–1^). We therefore conclude that this pathway is not accessible under our adiabatically cooled gas-phase conditions. At the same time, we notice that this pathway might come into play under room-temperature solution conditions, where much higher internal energies are available and where generally excitation at the absorption maximum takes place.

Finally, we consider intersystem crossing from the V(ππ*) state to the triplet manifold. We model such a process using semiclassical Marcus theory, from which the ISC rate can be calculated^53^ as follows:
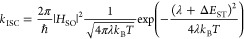
2where *H*_SO_ is the spin–orbit coupling matrix element between singlet and triplet states under consideration, λ is the reorganization energy, and Δ*E*_ST_ is the energy gap between the optimized singlet and triplet states involved in the ISC process (Figure S5), while *k*_B_ and *T* denote the Boltzmann constant and absolute temperature, respectively. λ corresponds to the energy difference between the triplet state energy at the optimized V(ππ*) geometry () and the energy of the optimized triplet state (). In the present case, several potential final triplet states *T*_*n*_ need to be considered, which include the *T*_1_ (V(^3^ππ*)), *T*_2_ (V’(^3^ππ*)), and *T*_5_ (^3^nπ*) states of which the equilibrium geometries are shown in Figure S5. Apart from these states, two more ππ* triplet states fall within the relevant energy range designated as *T*_3_(^3^ππ*) and *T*_4_(^3^ππ*). Geometry optimization of the *T*_3_(^3^ππ*) state was not possible as root-switching with the V’(^3^ππ*) state occurred. Geometry optimization of the *T*_4_(^3^ππ*) state, on the other hand, led to the structure depicted in Figure S5.

Table 4 reports for each of these states the parameters needed to calculate the ISC rate from S_1_ to the pertinent triplet state. In order to assess the geometry dependence of the SOC matrix element, we have calculated *H*_SO_ at both the equilibrium geometry of S_1_ as well as at the equilibrium geometry of *T*_*n*_. Table 4 shows that for the majority of the ISC pathways, there is indeed a non-negligible geometry dependence of *H*_SO_ and *k*_ISC_. In order to obtain the correct ISC rate, one would therefore need to incorporate this geometry dependence in the coupling matrix element between the initial and final Born–Oppenheimer states, which would exclude a semiclassical Marcus theoretical approach. In the following, we will therefore base our discussion on the predicted ISC rates, assuming that the appropriately calculated rate would be in the range of values found by the calculations at the equilibrium geometry of S_1_ and *T*_*n*_.

**Table 4:** Marcus Parameters and ISC Rate for  Transition Based on eq 2^a^^b^

State	⟨V(ππ*)|*H*_SO_|*T_n_*⟩ (cm^−1^)	(eV)	λ (eV)	(ns)	R2PI τ_1_ (ns)
*T*_4_	0.2 (0.2)	0.292	0.288	-	-
*T*(nπ*)	21.9 (13.9)	0.249	0.739	10^18^ (10^18^)	-
*T*_2_	0.4 (0.09)	0.177	0.237	4 (83)	3.2
*T*_1,tw_	2.9 (0.08)	1.432	–0.033	-	-

a Energies were calculated with DFT/MRCI.

b SOCMEs and rates computed at 10 K, evaluated at V(ππ*) equilibrium geometry; within parentheses: corresponding numbers evaluated at *T*_*n*_ equilibrium geometry.

As expected on the basis of El-Sayed’s rules for intersystem crossing,^54^ the spin–orbit coupling between the V(ππ*) and ^3^nπ* states is relatively large. The ISC rate between these two states is nevertheless found to be very small, because of the large difference between Δ*E*_ST_ and the reorganization energy λ. On the other hand, despite the small spin–orbit coupling between the V(ππ*) and *T*_2_ states, a relatively large rate is found for transitions between these two states. As a result of the distortion of the *T*_2_ state along a symmetry-lowering hydrogen out-of-plane (HOOP) coordinate (see side view of *T*_2_ in Figure S5), the V(ππ*) → *T*_2_ transition is associated with a large λ that compensates for the large Δ*E*_ST_. Table 4 shows that based on the calculated parameters, an ISC rate of (4–80 ns)^−1^ is predicted, which is in reasonable agreement with the pump–probe lifetime of 3.2 ns measured for the *syn/cis* conformer. The same table shows that for the remaining two ISC channels to the *T*_1_(V(^3^ππ*)) and *T*_4_(^3^ππ*) states, extremely small ISC rates are predicted. We thus come to the conclusion that ISC between the S_1_ V(ππ*) and *T*_2_ states is by far the dominant relaxation channel among the possible singlet–triplet relaxation channels with a rate that is in quantitative agreement with our experimental observations.

We conclude this part of the discussion with a further comment on the differences between the pump–probe traces of the *syn/cis* and *syn/trans* conformers and the pump–probe trace of the *anti/trans* conformer (Figure 5). The latter distinguishes itself by the small contribution of τ_1_ compared with the former ones. Since τ_1_ is similar in all cases, this excludes an explanation based on differences in the ISC rate. An alternative explanation might be that contributions from radiative or nonradiative transitions from V(ππ*) to *S*_0_ are different. The observation that similar oscillator strengths are calculated for the V(ππ*) → *S*_0_ transition of each conformer (0.67 for *syn/cis* and *anti/trans*, 0.68 for *anti/cis*, and 0.66 for *syn/trans*) suggests that for the *anti/trans* conformer, the V(ππ*) → *S*_0_ nonradiative decay rate is significantly smaller than for the other two conformers.

After the ISC of V(ππ*) to *T*_2_, rapid internal conversion to the lowest excited triplet state *T*_1_ takes place. The final step that brings the molecule back to its electronic ground state is the spin-forbidden *T*_1_ → *S*_0_ transition, which occurs at a rate of (30 ns)^−1^. We now aim to understand how this process can be relatively fast and why it is similar for all of the cinnamate-based systems studied so far. To this purpose, we consider once again the expression for the ISC rate derived from semiclassical Marcus theory (*vide supra*). Our calculations find—analogous to previous calculations on other cinnamate-based systems^8^—an MESX between *T*_1_ and *S*_0_. Under such conditions, the exponential term in the semiclassical Marcus theory expression can be replaced with the activation energy Δ*E*^‡^—the energy difference between the *T*_1_/*S*_0_ MESX and the energy  of *T*_1_ at its equilibrium geometry (see Figure S6)—leading to the expression:
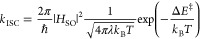
3One further difference with the original expression is that  is now computed at the *T*_1_/*S*_0_ MESX geometry.

**Figure 7:**
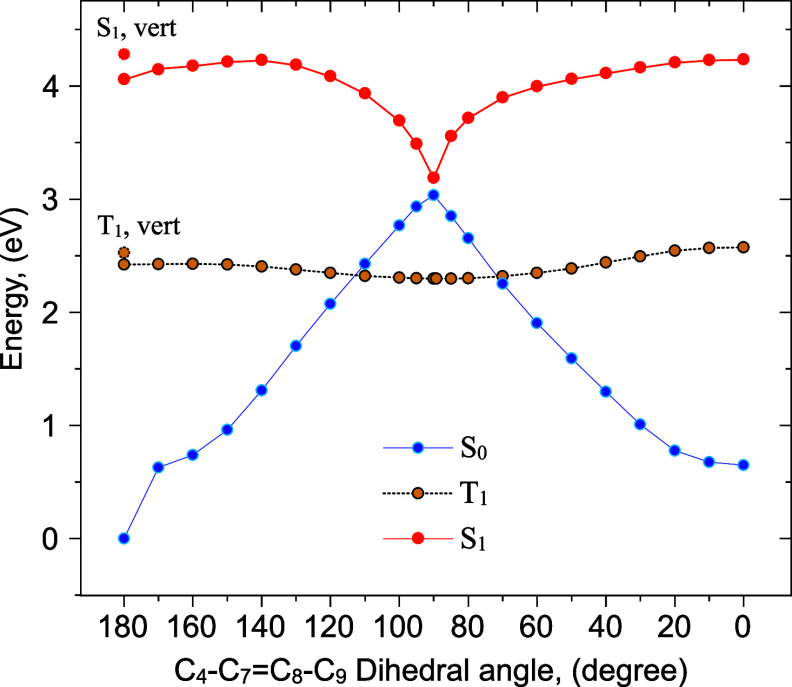
uDFT relaxed *T*_1_ scan. Corresponding single-point singlet energies were computed with TDDFT at *T*_1_ relaxed geometries. MESX structures appear on both sides of the twisted *T*_1_ minimum, one to the *E* isomer (reactant) and one to the *Z* isomer (product).

Figure 7 displays the relaxed *T*_1_ surface scan along the C_4_—C_7_=C_8_—C_9_ dihedral angle coordinate. Analogous to the S_1_/*S*_0_ MESX, the *T*_1_/*S*_0_ MESX structures directed to the *E* (reactant) and *Z* (photoproduct) isomers are characterized by a twist of the vinyl double bond (Figure S6), although to a lesser extent than found for the S_1_/*S*_0_ MESX. The relevant parameters and computed *T*_1_ → *S*_0_ ISC rates *k*_ISC_ are given in Table 5. Under our experimental conditions, energy is conserved, implying that after ISC from the V() state to the triplet manifold, *T*_1_ is populated with an excess energy of 1.6 eV. Following the equipartition theorem, such an excess energy corresponds to a temperature of 252 K. In combination with small Δ*E*^‡^ values, this leads to a rate of (5.0 ns)^−1^, which—considering the sensitivity of the rate to the calculated parameters in Equation 3—agrees quite well with the experimentally observed rate of (29 ns)^−1^.

**Table 5:** Marcus Parameters for  ISC (Equation 3)^a^^b^^c^

	⟨*T*_1_|*H*_SO_|*S*_0_⟩ (cm^−1^)	Δ*E*^‡^ (eV)	λ (eV)	(*k*_ISC_)^−1^ (ns)	R2PI τ_2_ (ns)
MESX,*E*	1.3	0.030	3.012	13.6	29
MESX,*Z*	1.3	0.019	2.875	8.0	

a SOCMEs evaluated at the uDFT-optimized MESX geometries with TD-DFT energies.

b Δ*E*^‡^ is based on the energy difference between the uDFT-optimized MESX and uDFT-optimized *T*_1_ geometries.

c Rates were computed at 252 K based on an excess energy of 1.6 eV.

**Figure 8:**
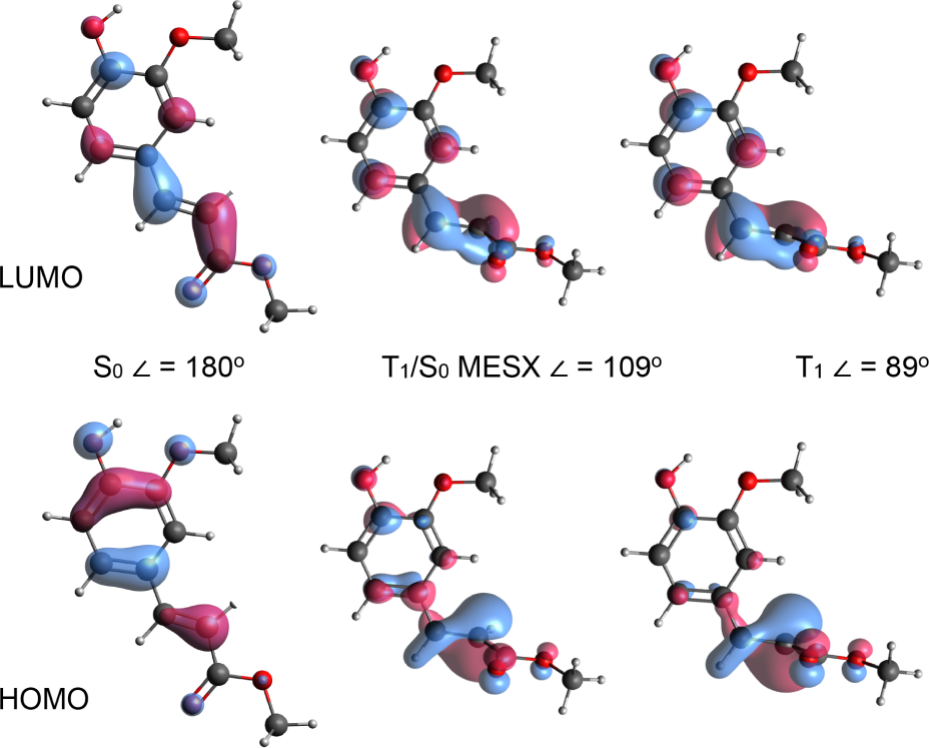
HOMO and LUMO orbitals of the *syn/cis* conformer of MF at *S*_0_, *T*_1_/*S*_0_ MESX, and *T*_1_ optimized geometries. Indicated for each geometry is the C_4_—C_7_=C_8_—C_9_ dihedral angle.

The observation that in compounds based on cinnamate, coumarate, ferulate, and sinapate chromophores, very similar *T*_1_ lifetimes that are found may now be rationalized by noting that in all these molecules, *T*_1_ is the V(^3^ππ*) state, which is at a similar energy and characterized by an equilibrium geometry in which the initial vinyl double bond is considerably twisted (Table 6). In order to obtain comparable *k*_ISC_ rates, however, Δ*E*^‡^ and hence the *T*_1_/*S*_0_ MESX energies should be similar. Inspection of the HOMO and LUMO orbitals at the *T*_1_ and *T*_1_/*S*_0_ MESX geometries (Figure 8) shows that at both geometries, the dominant part of the electron density is localized on the but-2-enoate part of the molecule. As a result, the substitution pattern on the phenyl ring is expected to have a minor influence on the energies of the *T*_1_ state at these geometries.

**Table 6:** Experimentally Observed *T*_1_ Lifetimes () and uDFT *T*_1_ Energies of MC, MF, and MS Calculated Here for MF or Extracted from References as Indicated in the Table for MC and MS^a^

Compound	(ns)	*T*_1_ (eV)
MC	29^36^	2.207/2.219^10^
MF	29	2.298
MS	27^29^	2.107/2.123^b^^37^

a The two entries for MC and MS refer to the energies of the two energetically close geometrical *T*_1_ structures that were reported in these references.

b Calculation was based on sinapic acid.

## 4 Conclusions

Methyl ferulate can be considered as a prototypical compound between coumarates and sinapates, two classes of nature-based UV filters with critically different electronically excited state manifolds. Studies of its electronically excited states and their dynamics are thus paramount to furthering our understanding of the changes imparted by substitutions of the cinnamate backbone. Furthermore, to connect to their real-life application, it is crucial to understand to what extent interactions with a solvent influence its properties.

In the present studies, such information has been obtained by applying Resonance-Enhanced MultiPhoton Ionization spectroscopic techniques on isolated and microsolvated compounds under molecular beam conditions in combination with quantum chemical calculations. Our studies demonstrate that the UV absorbing properties of MF are dominated by a bright  state. Importantly, experiment and theory show that the intensity distribution over the characteristic vibronic progression of an in-plane bending mode is highly conformation dependent. We find that water preferentially coordinates to the carbonyl group and in doing so, it red-shifts the bright  state. Interestingly, depletion-IR measurements lead to the conclusion that clusters are also generated in which water disrupts the intramolecular phenolic hydrogen bond, resulting in a blue-shift of the phenolic OH stretch mode. Such clusters have considerably higher energy but appear to be kinetically trapped during the expansion.

**Figure 9:**
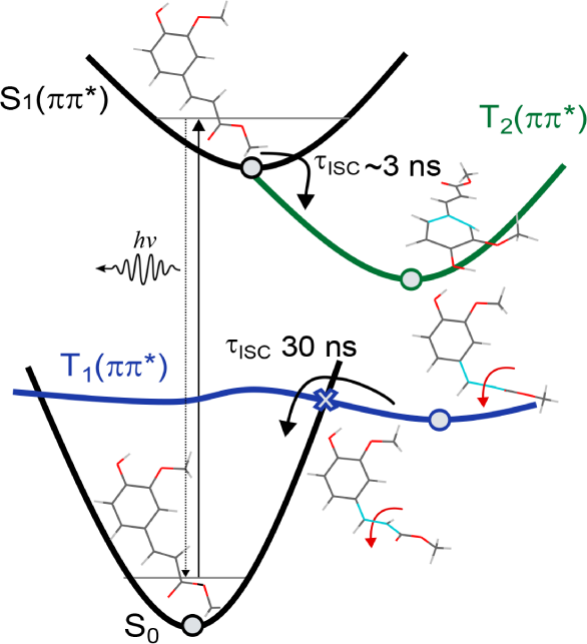
Summary of the photophysical decay pathway of adiabatically excited MF. Geometries of relevant optimized structures are shown as well. Note the cyan coloring of some atoms to highlight certain molecular distortions. ISC to the *T*_2_ state is followed by rapid internal conversion to *T*_1_. A twisting motion forms a *T*_1_/*S*_0_ MESX, which leads to ISC returning to the ground state.

Our studies show that for low internal energies in the  state, the absorbed photon energy is dissipated along the routes summarized in Figure 9. Similar to MS, the electron-donating ortho-methoxy group in MF raises the energy of the π-orbital, resulting in an adiabatic excited-state ordering that places the  state below the ^1^nπ* state. As a result, the triplet manifold is populated through El-Sayed-forbidden  ISC, in contrast to MC, where rapid El-Sayed-allowed ^1^nπ*  ISC takes place.^32^ Fast ISC back to *S*_0_ is made possible through a *T*_1_/*S*_0_ crossing-mediated route. Importantly, because the electron density is dominantly localized at the but-2-enoate tail for transient structures close to the *T*_1_/*S*_0_ crossing, very similar *T*_1_ decay rates are observed for cinnamates and phenyl-substituted cinnamates, irrespective of the further substitution details. The initial slow ISC from the  state in ferulates and sinapates is beneficial from a molecular heater point of view as triplet states are typically associated with photochemical degradation reactions. An additional advantage is that these compounds also have an efficient pathway of repopulating the ground state from *T*_1_.

The detailed insight that has been afforded by the present studies on the decay channels of electronically excited states in MF and related compounds breaks new ground for research into the design of highly efficient sunscreens and molecular heaters. Based on the present studies, a number of potentially interesting substitutions can be thought of that are currently further explored experimentally both under molecular beam as well as solution conditions.

## Supporting Information Available

Additional computational results of adiabatic and vertical excitation energies at various levels, description of lower electronically excited singlet states, and relative energies of MF conformers. Additional computational results on OH stretch frequencies and S_1_ excitation energies of MF-H_2_O conformers. Predicted *S*_0_ excitation spectrum of *anti/cis* conformer of MF. Comparison of UV–UV depletion spectra of *syn/cis* (a), *syn/trans* (c), and *anti/trans* (e) conformers of MF, and TD-DFT-computed vibrationally resolved excitation spectra of their V’ state. IR–UV depletion spectra in the OH stretch region of MF conformers. Monoexponential fit decay of the S_1_ vibrationless level *syn/cis* conformer. Calculated equilibrium geometries of lower-lying triplet states *syn/cis* MF and *T*_1_/*S*_0_ MESX structures of *E* (reactant) and *Z* (photoproduct) MF isomers. Coordinates of various structures of MF and MF-H_2_O discussed in the text (PDF)
