# Supplementary material for: Spectroscopy and Excited-State Dynamics of Methyl Ferulate in Molecular Beams
Source: J Phys Chem A. 2024 Dec 17;129(1):36–49. doi: 10.1021/acs.jpca.4c05792 (PMC11726680; doi:10.1021/acs.jpca.4c05792)
Supplement: Supplementary file 1 — jp4c05792_si_001.pdf [file jp4c05792_si_001.pdf]

Supporting information for: Spectroscopy and Excited-State Dynamics of Methyl Ferulate in Molecular Beams

Ivan Romanov,[†,△] Yorrick Boeije,[†,‡,¶,△] Josene M. Toldo,[§,‖] Mariana Telles do Casal,[§,⊥] Mario Barbatti,[§,#] and Wybren Jan Buma[*,†,@]

† *Van 't Hoff Institute for Molecular Sciences, University of Amsterdam, Science Park 904, 1098 XH Amsterdam, The Netherlands*

‡ *Department of Chemical Engineering and Biotechnology, University of Cambridge, Philippa Fawcett Drive, Cambridge CB3 0AS, U.K.*

¶ *Department of Physics, Cavendish Laboratory, University of Cambridge, JJ Thomson Avenue, Cambridge CB3 0HE, U.K*

§ *Aix Marseille University, CNRS, ICR, 13397 Marseille, France*

‖ *UCBL, ENS de Lyon, CNRS, LCH, UMR 5182, 69342, Lyon cedex 07, France*

⊥ *Department of Chemistry, Physical Chemistry and Quantum Chemistry Division, KU Leuven, 3001 Leuven, Belgium*

\# *Institut Universitaire de France, 75231 Paris, France*

@ *Radboud University, Institute for Molecules and Materials, FELIX Laboratory, Toernooiveld 7c, 6525 ED Nijmegen, The Netherlands*

△ *These authors contributed equally to this work.*

E-mail: w.j.buma@uva.nl

Supporting Information Available

Table S1: ωB97XD/aug-cc-pVTZ TD-DFT adiabatic and vertical excitation energies (eV) of transitions to lower electronically excited singlet states of methyl ferulate conformers with oscillator strength f given in parentheses.

Conformer	$S_1(V(\pi\pi^*))$	$S_2(V'(\pi\pi^*))$	S_3 (nπ^*)
syn/cis	4.06 / 4.28 (0.63)	4.67 / 4.81 (0.09)	4.41 / 5.02 (1×10^{-4})
anti/cis	4.02 / 4.29 (0.63)	4.67 / 4.74 (0.01)	- / 5.01 (1×10^{-4})
syn/trans	4.08 / 4.30 (0.62)	4.68 / 4.81 (0.10)	4.52 / 5.02 (1×10^{-4})
anti/trans	4.05 / 4.31 (0.63)	4.67 / 4.75 (0.01)	4.50 / 5.00 (1×10^{-4})

Table S2: BH-LYP/def2-TZVP DFT/MRCI vertical excitation energies (eV) of transitions to lower electronically excited singlet states of methyl ferulate conformers with oscillator strength f given in parentheses. S_0 geometry optimized at ωB97XD/cc-pVDZ DFT level.

Conformer	State	Energy (eV)	Orbital Occupation
syn/cis	S_1 (V($\pi\pi^*$))	4.02 (0.56)	LUMO 0.88 — HOMO 1.09
	S_2 (V'($\pi\pi^*$))	4.40 (0.25)	LUMO+1 0.69/0.33 — HOMO-1 1.50/1.48
	S_3 (nπ^*)	4.45 (4×10^{-4})	LUMO 0.88 — HOMO-3 1.01
anti/cis	S_1 (V($\pi\pi^*$))	4.07 (0.66)	LUMO 0.97 — HOMO 1.09
	S_2 (V'($\pi\pi^*$))	4.27 (0.05)	LUMO+1 0.54/0.50 — HOMO-1 1.46/1.51
	S_3 (nπ^*)	4.46 (4×10^{-4})	LUMO 0.90 — HOMO-3 1.01
syn/trans	S_1 (V($\pi\pi^*$))	4.05 (0.56)	LUMO 0.87 — HOMO 1.10
	S_2 (nπ^*)	4.33 (4×10^{-4})	LUMO 0.91 — HOMO-3 1.01
	S_3 (V'($\pi\pi^*$))	4.42 (0.28)	LUMO+1 0.70/0.32 — HOMO-1 1.49/1.50
anti/trans	S_1 (V($\pi\pi^*$))	4.10 (0.65)	LUMO 0.97 — HOMO 1.05
	S_2 (V'($\pi\pi^*$))	4.28 (0.07)	LUMO+1 0.54/0.50 — HOMO-1 1.44/1.53
	S_3 (nπ^*)	4.42 (4×10^{-4})	LUMO 0.93 — HOMO-3 1.01

Table S3: BH-LYP/def2-TZVP DFT/MRCI vertical and adiabatic excitation energies (eV) of methyl ferulate conformers at optimized V, V' and nπ^* geometries. Optimized state marked with dagger.

Conformer	S_0	V	V'	nπ^*
syn/cis				
S_1	4.02 (V)	3.81 (V)†	3.94 (V)	3.83 (nπ^*)†
S_2	4.40 (V')	4.24 (nπ^*)	4.22 (V')†	4.27 (V)
S_3	4.45 (nπ^*)	4.30 (V')	4.33 (nπ^*)	4.73 (V')
anti/cis				
S_1	4.07 (V)	3.83 (V)†	3.99	-
S_2	4.27 (V')	4.24 (V')	4.12 (V')†	-
S_3	4.45 (nπ^*)	4.30 (V')	4.33 (nπ^*)	-
syn/trans				
S_1	4.05 (V)	3.85 (V)†	3.97 (V)	3.78 (nπ^*)†
S_2	4.33 (V')	4.18 (nπ^*)	4.25 (nπ^*)	4.32 (V)
S_3	4.42 (nπ^*)	4.33 (V')	4.25 (V')†	4.74 (V')
anti/trans				
S_1	4.10 (V)	3.86 (V)†	4.01 (V)	3.76 (nπ^*)†
S_2	4.28 (V')	4.17 (nπ^*)	4.14 (V')†	4.33 (V)
S_3	4.42 (nπ^*)	4.25 (V')	4.32 (nπ^*)	4.68 (V')

Table S4: Relative ωB97XD/cc-pVDZ DFT energies (kcal/mol) and corresponding Boltzmann population of MF-H_2O conformers.

Conformer	ΔE_{carb}	RT Pop.	ΔE_{phen}	RT Pop.	ΔE_{phen2}	RT Pop.
syn/cis	0.23	0.68	1.44	0.09	6.57	2×10^{-5}
anti/cis	0	1.00	1.57	0.07	6.19	3×10^{-5}
syn/trans	0.47	0.46	2.07	0.03	7.00	7×10^{-6}
anti/trans	1.68	0.06	2.58	0.01	7.45	3×10^{-7}

Table S5: ωB97XD/cc-pVDZ DFT calculated OH stretch frequencies (cm^{-1}) for MF-H_2O clusters. ν_1 represents the hydrogen-bonded OH stretch of coordinated H_2O, ν_2 the PhOH stretch, and ν_3 the non-hydrogen-bonded OH stretch of coordinated H_2O. Intensity (a.u.) of vibrational transition given in parentheses.

DFT Conformer	ν_1	ν_2	ν_3
anti/cis_carb	3545.61 (30)	3626.82 (23)	3721.43 (7)
syn/cis_carb	3549.32 (48)	3625.62 (33)	3723.77 (12)
anti/trans_carb	3543.28 (37)	3628.60 (25)	3725.56 (8)
syn/trans_carb	3535.82 (34)	3625.66 (22)	3722.92 (7)
anti/cis_phen	3339.33 (100)	3583.26 (30)	3725.97 (12)
syn/cis_phen	3343.87 (100)	3586.67 (30)	3728.22 (12)
anti/trans_phen	3338.85 (100)	3585.20 (29)	3725.98 (12)
syn/trans_phen	3338.35 (100)	3585.52 (30)	3727.51 (12)
anti/cis_phen2	3633.33 (42)	3695.11 (33)	3740.21 (25)
syn/cis_phen2	3632.75 (42)	3695.98 (33)	3740.07 (25)

Table S6: BH-LYP/def2-TZVP DFT/MRCI vertical excitation energies (eV) of transitions to lower electronically excited singlet states of MF-H_2O) conformers with oscillator strength f given in parentheses.

Conformer	$S_1(V(\pi\pi^*))$, eV
syn/cis_carb	3.75 / 3.94 (0.65)
anti/cis_carb	3.73 / 3.96 (0.70)
syn/trans_carb	3.78 / 3.95 (0.61)
anti/trans_carb	3.77 / 3.98 (0.67)
syn/cis_phen	3.77 / 3.99 (0.59)
anti/cis_phen	3.79 / 4.06 (0.73)
syn/trans_phen	3.81 / 4.01 (0.56)
anti/trans_phen	3.83 / 4.09 (0.72)
syn/cis_phen2	3.83 / 4.08 (0.53)
anti/cis_phen2	3.83 / 4.04 (0.45)
syn/trans_phen2	3.86 / 4.06 (0.42)
anti/trans_phen2	3.86 / 4.11 (0.48)

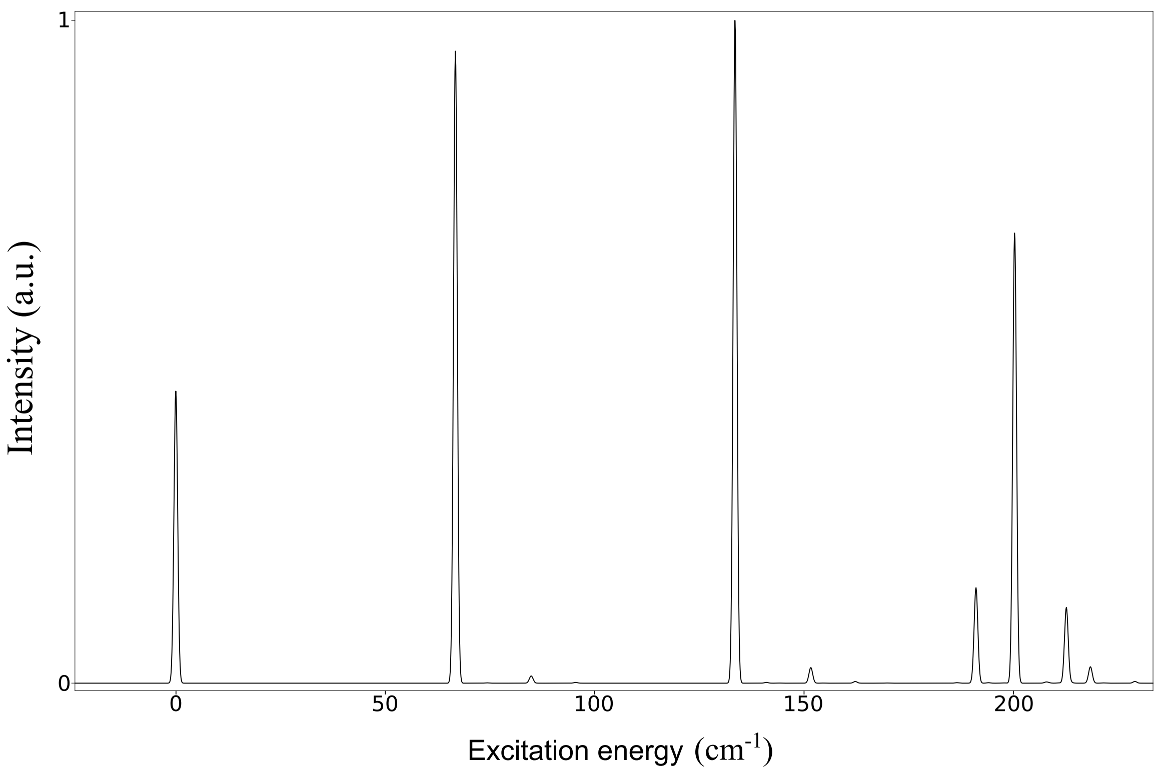

Figure S1: Low-energy part of computed $V(\pi\pi^*) \leftarrow S_0$ excitation spectrum of *anti/cis* conformer of methyl ferulate showing dominant 'quartet'-like vibrational progression pattern. Excitation energy relative to excitation energy of 0-0 transition.

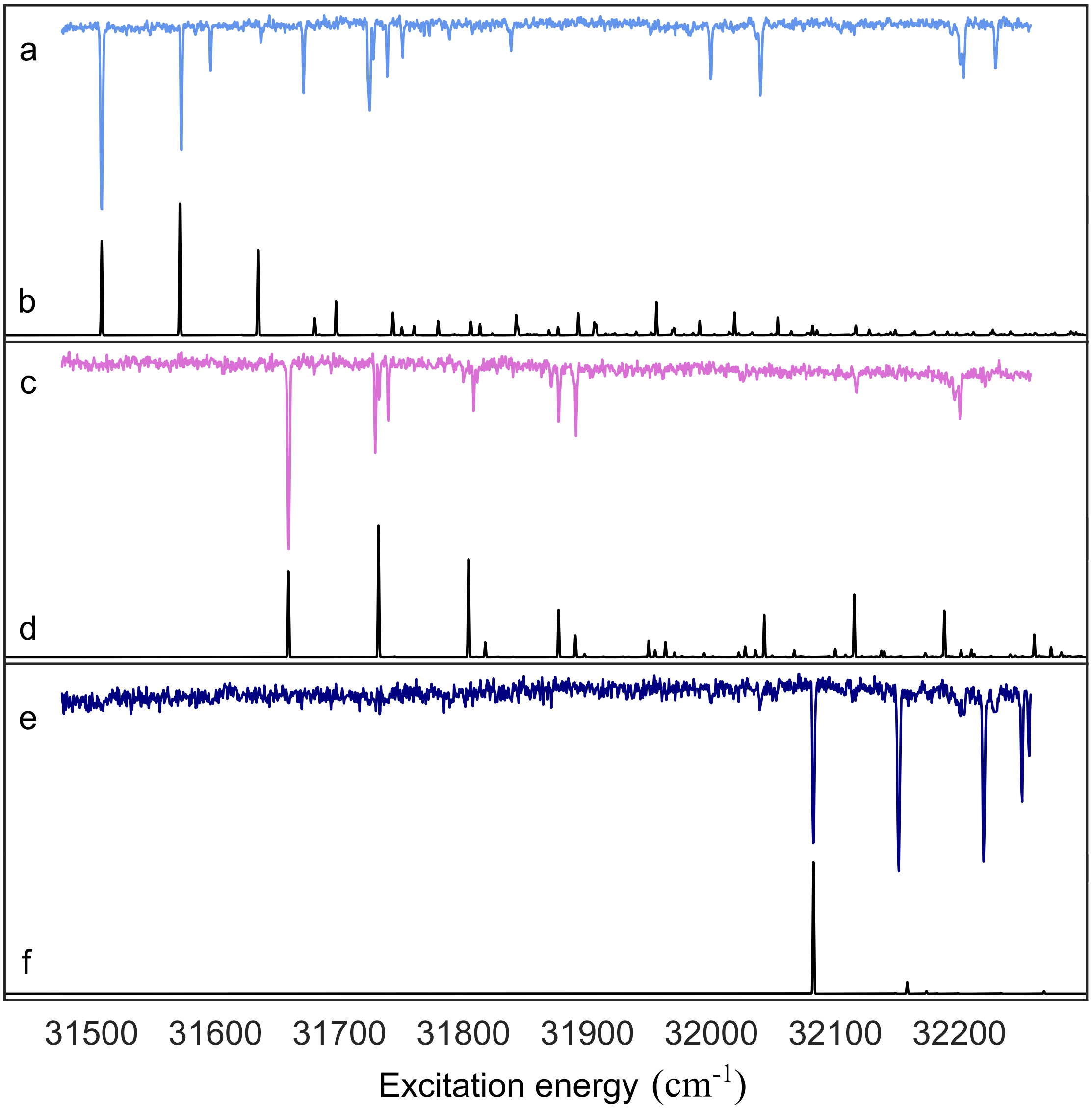

Figure S2: Comparison between the experimentally recorded UV-UV depletion spectra of *syn/cis* (a), *syn/trans* (c) and *anti/trans* (e) conformers of MF, and TD-DFT computed vibrationally-resolved excitation spectra of their V' state shown in (b), (d), and (f).

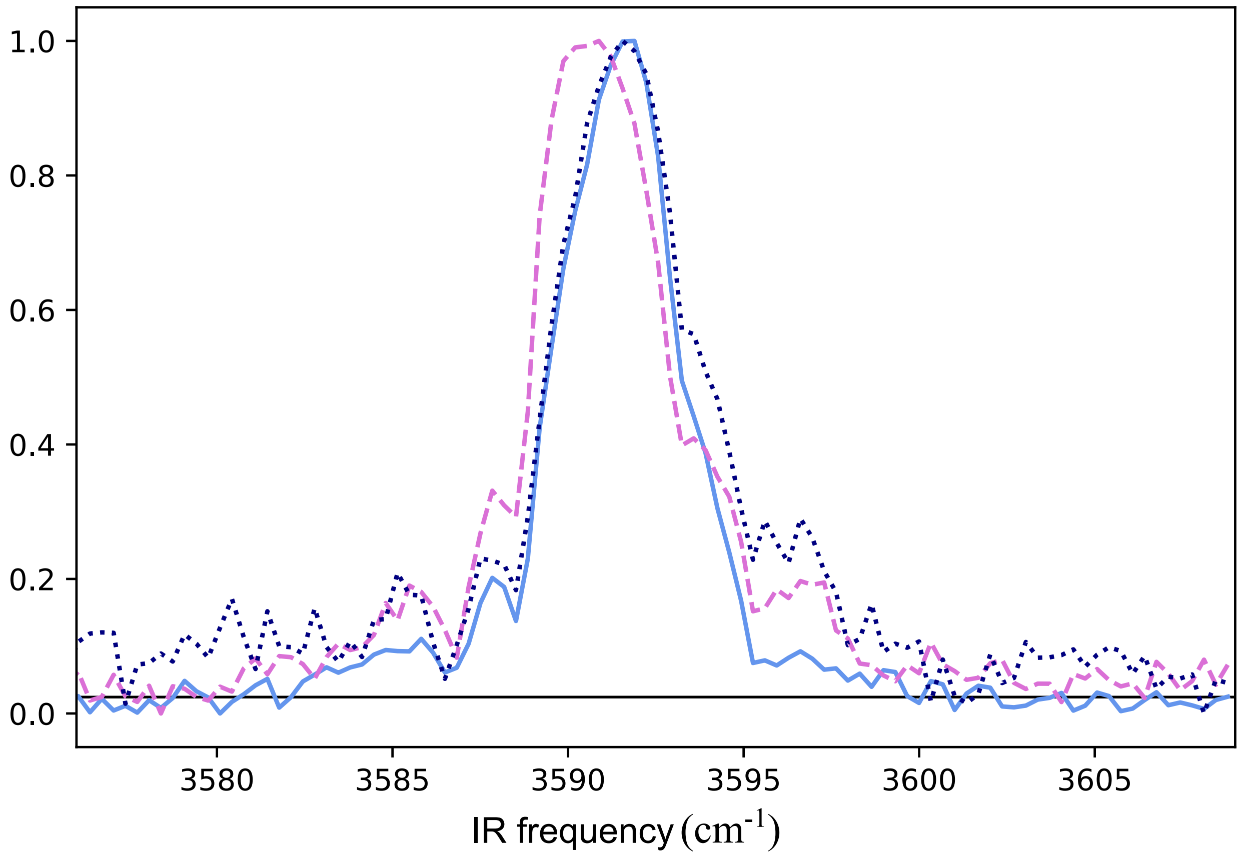

Figure S3: Experimentally recorded IR-UV depletion spectra of the OH-stretch region of *syn/cis* (light blue solid line), *syn/trans* (pink dashed line) and *anti/trans* (dark blue dotted line) conformers of MF.

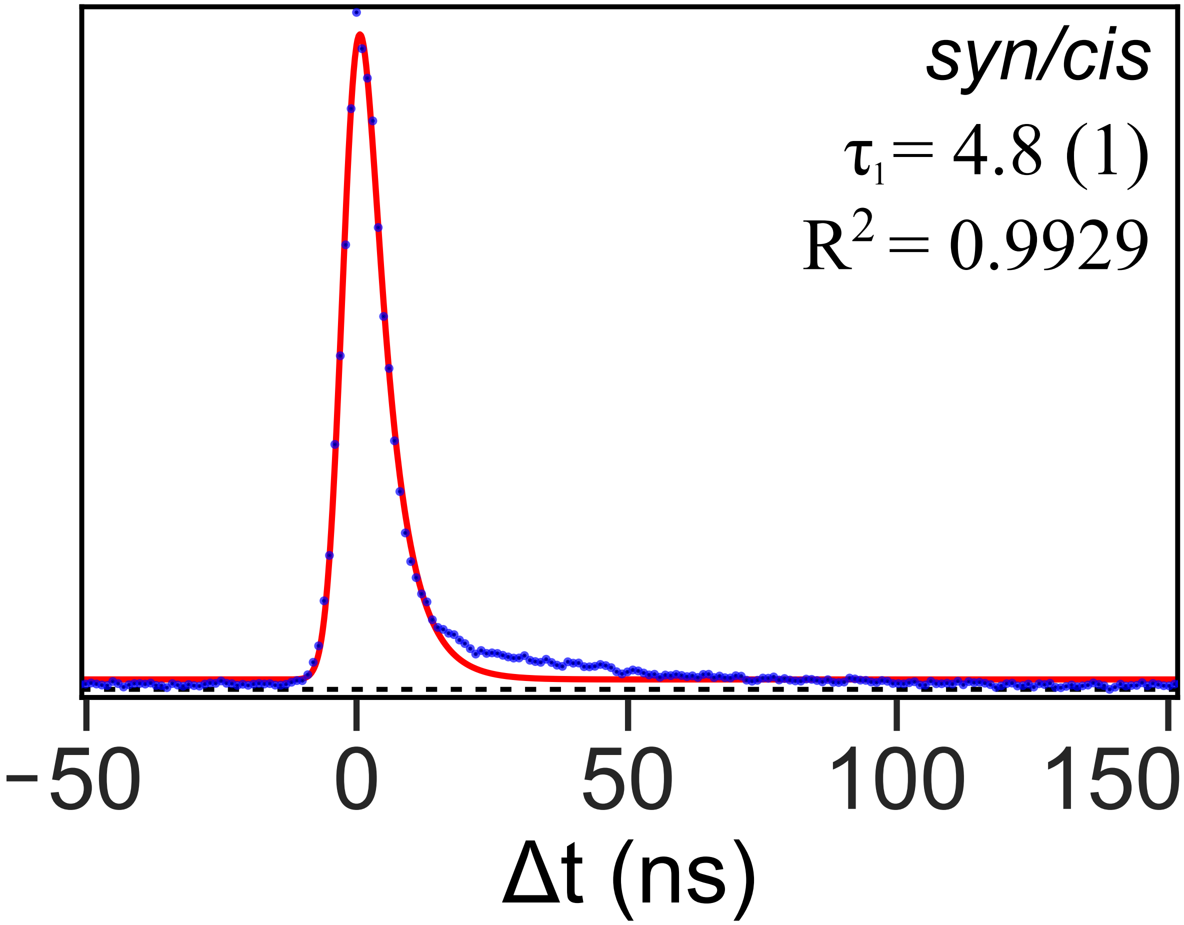

Figure S4: Time-resolved (1+1') R2PI decay curve after excitation of the vibrationless level of the $V(\pi\pi^*)$ state of the *syn/cis* conformation of methyl ferulate taken with time steps of 1 ns. The solid lines is a mono-exponential fit convoluted with a Gaussian profile with decay time in ns.

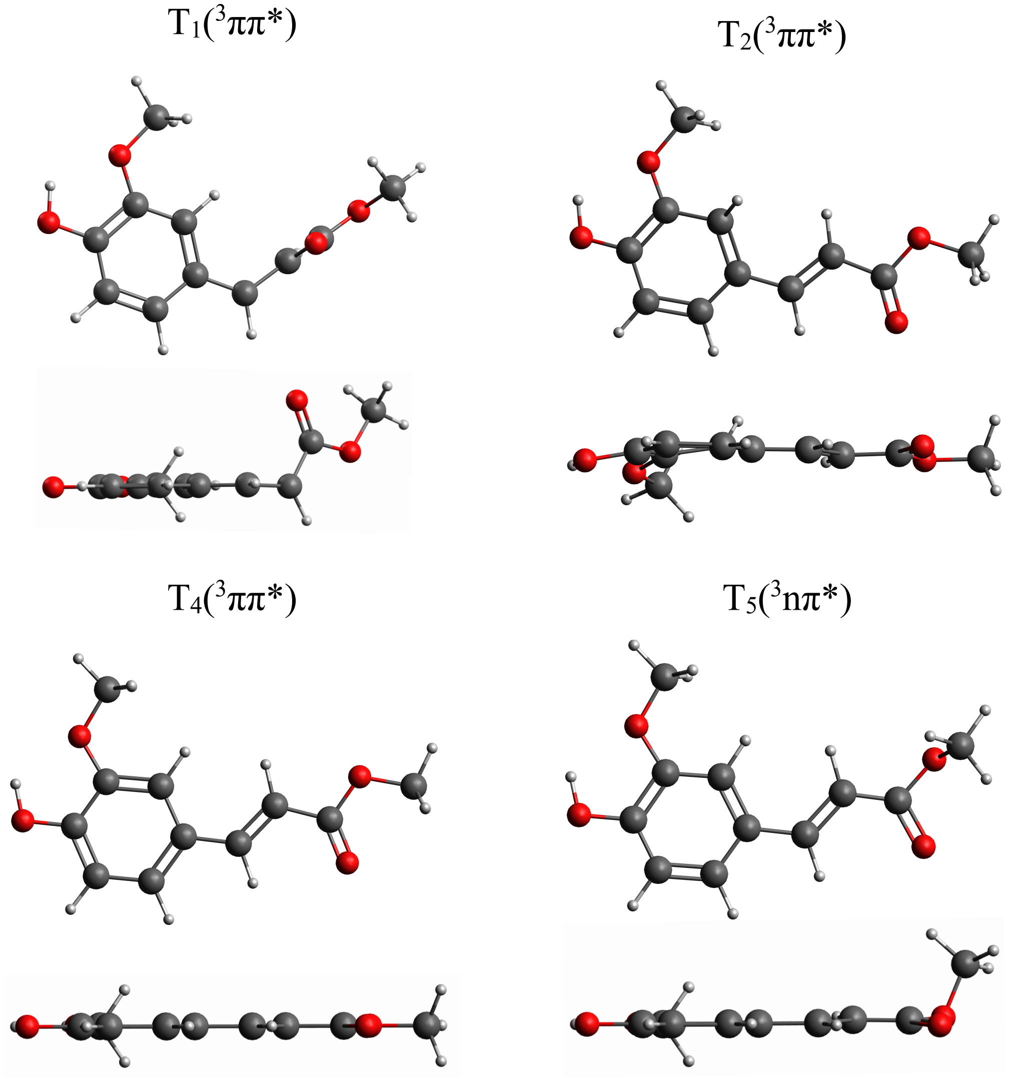

Figure S5: Equilibrium geometries of triplet states used to estimate ISC rates.

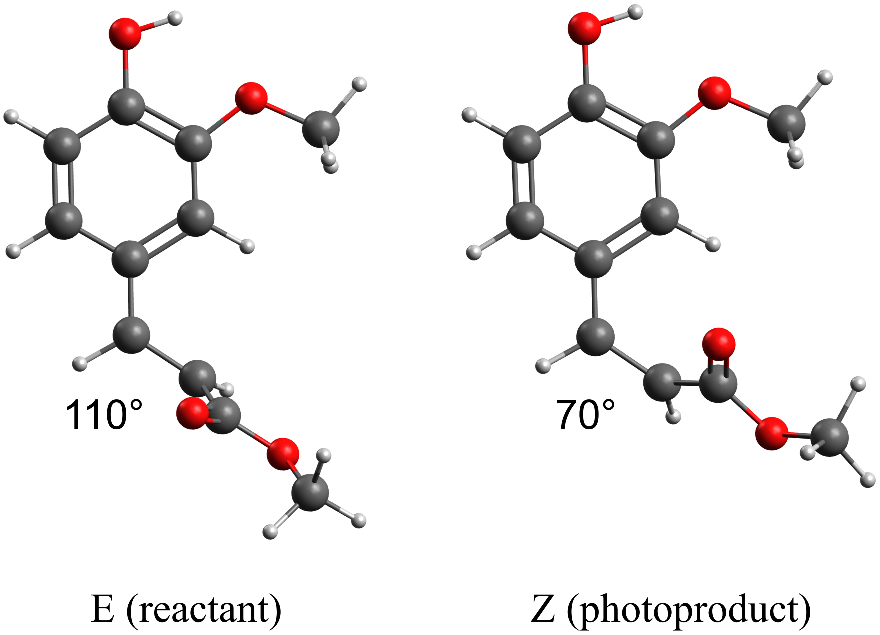

Figure S6: T_1/S_0 MESX structures of the E (reactant) and Z (photoproduct) isomers. Indicated values are the C_4-C_7=C_8-C_9 dihedral angles.

Table S7: Equilibrium geometry of methyl ferulate *syn/cis* conformer S_0 state.

Atom	X	Y	Z
C	1.136173	0.049601	0.000000
C	2.404828	0.599072	0.000000
C	2.580074	2.001291	0.000000
C	1.466308	2.827755	0.000000
C	0.187644	2.270343	0.000000
C	0.000000	0.885949	0.000000
C	-1.362515	0.353932	0.000000
C	-1.743203	-0.933388	0.000000
C	-3.180238	-1.271200	0.000000
O	-3.356623	-2.608047	0.000000
C	-4.714264	-3.038484	0.000000
O	-4.098751	-0.482665	0.000000
O	3.824538	2.521753	0.000000
O	3.583096	-0.090764	0.000000
C	3.523826	-1.501001	0.000000
H	4.439053	1.773399	0.000000
H	4.561952	-1.852574	0.000000
H	3.010262	-1.879920	0.899771
H	3.010262	-1.879920	-0.899771
H	1.010301	-1.032293	0.000000
H	-0.683492	2.928153	0.000000
H	-2.168879	1.095273	0.000000
H	-1.042888	-1.769996	0.000000
H	-4.681046	-4.133732	0.000000
H	-5.241046	-2.670686	0.892444
H	1.616690	3.907539	0.000000
H	-5.241046	-2.670686	-0.892444

S_0 (ωB97XD/cc-pVDZ) DFT Energy = -727.088975639 Hartree

Table S8: Equilibrium geometry of methyl ferulate *syn/cis* conformer S_1 $V(\pi\pi^*)$ state.

Atom	X	Y	Z
C	1.085844	-0.009259	0.000000
C	2.389079	0.455683	0.000000
C	2.677302	1.846662	0.000000
C	1.620742	2.790275	0.000000
C	0.326349	2.347496	0.000000
C	0.000000	0.935968	0.000000
C	-1.353474	0.528547	0.000000
C	-1.775859	-0.806458	0.000000
C	-3.187700	-1.116999	0.000000
O	-3.407491	-2.461906	0.000000
C	-4.774448	-2.840073	0.000000
O	-4.100755	-0.302262	0.000000
O	3.946410	2.272802	0.000000
O	3.511245	-0.306549	0.000000
C	3.361191	-1.715681	0.000000
H	4.511398	1.482851	0.000000
H	4.375328	-2.129962	0.000000
H	2.822537	-2.053084	0.900053
H	2.822537	-2.053084	-0.900053
H	0.870255	-1.074700	0.000000
H	-0.498353	3.062577	0.000000
H	-2.135078	1.291827	0.000000
H	-1.077414	-1.644550	0.000000
H	-4.784704	-3.936850	0.000000
H	-5.293765	-2.454775	0.891095
H	1.871595	3.850879	0.000000
H	-5.293765	-2.454775	-0.891095

S_1 (ωB97XD/cc-pVDZ) DFT Energy = -726.936658500 Hartree

Table S9: Equilibrium geometry of methyl ferulate *syn/cis* conformer S_2 $V'(\pi\pi^*)$ state.

Atom	X	Y	Z
C	1.072428	0.010534	0.000000
C	2.433750	0.410814	0.000000
C	2.786876	1.776528	0.000000
C	1.751915	2.723126	0.000000
C	0.386465	2.335640	0.000000
C	0.000000	0.976943	0.000000
C	-1.357453	0.556424	0.000000
C	-1.807753	-0.748317	0.000000
C	-3.232909	-1.023259	0.000000
O	-3.477872	-2.362915	0.000000
C	-4.851766	-2.717111	0.000000
O	-4.130949	-0.198947	0.000000
O	4.082127	2.173055	0.000000
O	3.477847	-0.440245	0.000000
C	3.244215	-1.841209	0.000000
H	4.628431	1.375376	0.000000
H	4.234169	-2.309208	0.000000
H	2.691079	-2.146851	0.901606
H	2.691079	-2.146851	-0.901606
H	0.827841	-1.049901	0.000000
H	-0.381417	3.109314	0.000000
H	-2.124878	1.335394	0.000000
H	-1.144646	-1.614182	0.000000
H	-4.880914	-3.813474	0.000000
H	-5.364060	-2.323636	0.891101
H	2.023548	3.779390	0.000000
H	-5.364060	-2.323636	-0.891101

S_2 (ωB97XD/cc-pVDZ) DFT Energy = -726.915414484 Hartree

Table S10: Equilibrium geometry of methyl ferulate *syn/cis* conformer S_3 ($n\pi^*$) state.

Atom	X	Y	Z
C	1.119357	0.051703	0.000000
C	2.406288	0.560827	0.000000
C	2.634781	1.952193	0.000000
C	1.545759	2.812274	0.000000
C	0.249219	2.303298	0.000000
C	0.000000	0.919614	0.000000
C	-1.361470	0.438454	0.000000
C	-1.759281	-0.899395	0.000000
C	-3.081723	-1.287456	0.000000
O	-3.432314	-2.581249	0.000000
C	-4.821090	-2.919958	0.000000
O	-4.092175	-0.447380	0.000000
O	3.901944	2.433578	0.000000
O	3.560266	-0.175811	0.000000
C	3.447746	-1.580189	0.000000
H	4.486501	1.662553	0.000000
H	4.471685	-1.972287	0.000000
H	2.919743	-1.941739	0.899071
H	2.919743	-1.941739	-0.899071
H	0.966291	-1.026502	0.000000
H	-0.595018	2.995630	0.000000
H	-2.132550	1.211137	0.000000
H	-1.052106	-1.730195	0.000000
H	-4.848130	-4.014260	0.000000
H	-5.323149	-2.535526	0.900088
H	1.730848	3.887159	0.000000
H	-5.323149	-2.535526	-0.900088

S_3 (ωB97XD/cc-pVDZ) DFT Energy = -726.929494762 Hartree

Table S11: Equilibrium geometry of methyl ferulate *syn/trans* conformer S_0 state.

Atom	X	Y	Z
C	1.199961	-0.046555	0.000000
C	2.420160	0.603824	0.000000
C	2.481904	2.015647	0.000000
C	1.305019	2.749248	0.000000
C	0.075598	2.089872	0.000000
C	0.000000	0.695062	0.000000
C	-1.317382	0.055716	0.000000
C	-1.582470	-1.260696	0.000000
C	-2.945682	-1.824712	0.000000
O	-3.911413	-0.883280	0.000000
C	-5.243882	-1.384605	0.000000
O	-3.183088	-3.011607	0.000000
O	3.680435	2.634545	0.000000
O	3.649854	0.011521	0.000000
C	3.705128	-1.399322	0.000000
H	4.353418	1.938226	0.000000
H	4.768329	-1.665387	0.000000
H	3.224069	-1.818502	0.899782
H	3.224069	-1.818502	-0.899782
H	1.162654	-1.135019	0.000000
H	-0.845315	2.676215	0.000000
H	-2.171653	0.738804	0.000000
H	-0.802508	-2.023866	0.000000
H	-5.894150	-0.502684	0.000000
H	-5.431256	-1.998639	-0.892715
H	1.367172	3.837679	0.000000
H	-5.431256	-1.998639	0.892715

S_0 (ωB97XD/cc-pVDZ) DFT Energy = -727.087603730 Hartree

Table S12: Equilibrium geometry of methyl ferulate *syn/trans* conformer S_1 $V(\pi\pi^*)$ state.

Atom	X	Y	Z
C	1.150698	-0.123127	0.000000
C	2.417463	0.435072	0.000000
C	2.605701	1.843320	0.000000
C	1.483613	2.708319	0.000000
C	0.224970	2.172502	0.000000
C	0.000000	0.740777	0.000000
C	-1.324461	0.240345	0.000000
C	-1.643860	-1.122765	0.000000
C	-2.992250	-1.648579	0.000000
O	-3.937539	-0.663255	0.000000
C	-5.277855	-1.126205	0.000000
O	-3.288294	-2.833853	0.000000
O	3.840593	2.359931	0.000000
O	3.590518	-0.244641	0.000000
C	3.543465	-1.661635	0.000000
H	4.461233	1.612906	0.000000
H	4.585237	-2.000307	0.000000
H	3.031074	-2.037158	0.900115
H	3.031074	-2.037158	-0.900115
H	1.015587	-1.201396	0.000000
H	-0.648633	2.827051	0.000000
H	-2.146074	0.958392	0.000000
H	-0.873792	-1.895505	0.000000
H	-5.907796	-0.228326	0.000000
H	-5.486018	-1.738187	-0.891039
H	1.657000	3.784277	0.000000
H	-5.486018	-1.738187	0.891039

S_1 (ωB97XD/cc-pVDZ) DFT Energy = -726.934806315 Hartree

Table S13: Equilibrium geometry of methyl ferulate *syn/trans* conformer S_2 $V'(\pi\pi^*)$ state.

Atom	X	Y	Z
C	1.128167	-0.116518	0.000000
C	2.463773	0.361594	0.000000
C	2.737185	1.745334	0.000000
C	1.648640	2.629322	0.000000
C	0.307802	2.163734	0.000000
C	0.000000	0.785909	0.000000
C	-1.332036	0.287457	0.000000
C	-1.692830	-1.046144	0.000000
C	-3.063185	-1.523496	0.000000
O	-3.978808	-0.514300	0.000000
C	-5.332412	-0.937897	0.000000
O	-3.395088	-2.696078	0.000000
O	4.006886	2.217413	0.000000
O	3.554438	-0.428158	0.000000
C	3.401408	-1.840784	0.000000
H	4.599525	1.453574	0.000000
H	4.416620	-2.250960	0.000000
H	2.866654	-2.177354	0.901588
H	2.866654	-2.177354	-0.901588
H	0.946701	-1.189361	0.000000
H	-0.502578	2.892889	0.000000
H	-2.134186	1.028516	0.000000
H	-0.961944	-1.855763	0.000000
H	-5.934863	-0.021485	0.000000
H	-5.559769	-1.542209	-0.891198
H	1.858451	3.699624	0.000000
H	-5.559769	-1.542209	0.891198

S_2 (ωB97XD/cc-pVDZ) DFT Energy = -726.914035977 Hartree

Table S14: Equilibrium geometry of methyl ferulate *syn/trans* conformer S_3 ($n\pi^*$) state.

Atom	X	Y	Z
C	1.128167	-0.116518	0.000000
C	2.463773	0.361594	0.000000
C	2.737185	1.745334	0.000000
C	1.648640	2.629322	0.000000
C	0.307802	2.163734	0.000000
C	0.000000	0.785909	0.000000
C	-1.332036	0.287457	0.000000
C	-1.692830	-1.046144	0.000000
C	-3.063185	-1.523496	0.000000
O	-3.978808	-0.514300	0.000000
C	-5.332412	-0.937897	0.000000
O	-3.395088	-2.696078	0.000000
O	4.006886	2.217413	0.000000
O	3.554438	-0.428158	0.000000
C	3.401408	-1.840784	0.000000
H	4.599525	1.453574	0.000000
H	4.416620	-2.250960	0.000000
H	2.866654	-2.177354	0.901588
H	2.866654	-2.177354	-0.901588
H	0.946701	-1.189361	0.000000
H	-0.502578	2.892889	0.000000
H	-2.134186	1.028516	0.000000
H	-0.961944	-1.855763	0.000000
H	-5.934863	-0.021485	0.000000
H	-5.559769	-1.542209	-0.891198
H	1.858451	3.699624	0.000000
H	-5.559769	-1.542209	0.891198

S_3 (ωB97XD/cc-pVDZ) DFT Energy = -726.924985463 Hartree

Table S15: Equilibrium geometry of methyl ferulate *anti/cis* conformer S_0 state.

Atom	X	Y	Z
C	1.199961	-0.046555	0.000000
C	2.420160	0.603824	0.000000
C	2.481904	2.015647	0.000000
C	1.305019	2.749248	0.000000
C	0.075598	2.089872	0.000000
C	0.000000	0.695062	0.000000
C	-1.317382	0.055716	0.000000
C	-1.582470	-1.260696	0.000000
C	-2.945682	-1.824712	0.000000
O	-3.911413	-0.883280	0.000000
C	-5.243882	-1.384605	0.000000
O	-3.183088	-3.011607	0.000000
O	3.680435	2.634545	0.000000
O	3.649854	0.011521	0.000000
C	3.705128	-1.399322	0.000000
H	4.353418	1.938226	0.000000
H	4.768329	-1.665387	0.000000
H	3.224069	-1.818502	0.899782
H	3.224069	-1.818502	-0.899782
H	1.162654	-1.135019	0.000000
H	-0.845315	2.676215	0.000000
H	-2.171653	0.738804	0.000000
H	-0.802508	-2.023866	0.000000
H	-5.894150	-0.502684	0.000000
H	-5.431256	-1.998639	-0.892715
H	1.367172	3.837679	0.000000
H	-5.431256	-1.998639	0.892715

S_0 (ωB97XD/cc-pVDZ) DFT Energy = -727.088489850 Hartree

Table S16: Equilibrium geometry of methyl ferulate *anti/cis* conformer S_1 $V(\pi\pi^*)$ state.

Atom	X	Y	Z
C	1.142131	1.138802	0.000000
C	0.982560	2.505524	0.000000
C	-0.327432	3.102619	0.000000
C	-1.457490	2.281233	0.000000
C	-1.316414	0.909790	0.000000
C	0.000000	0.281300	0.000000
C	0.147171	-1.123761	0.000000
C	-0.924916	-2.027406	0.000000
C	-0.664192	-3.448065	0.000000
O	-1.818058	-4.174045	0.000000
C	-1.635144	-5.579800	0.000000
O	0.441270	-3.975015	0.000000
O	-0.444792	4.437078	0.000000
O	1.965108	3.432273	0.000000
C	3.311372	2.984845	0.000000
H	0.453859	4.804073	0.000000
H	3.931659	3.887452	0.000000
H	3.523064	2.386764	0.900383
H	3.523064	2.386764	-0.900383
H	2.133054	0.686189	0.000000
H	-2.203699	0.278211	0.000000
H	1.151922	-1.552647	0.000000
H	-1.967761	-1.708124	0.000000
H	-2.641686	-6.015723	0.000000
H	-1.078383	-5.909229	-0.891022
H	-2.440813	2.752676	0.000000
H	-1.078383	-5.909229	0.891022

S_1 (ωB97XD/cc-pVDZ) DFT Energy = -726.937541593 Hartree

Table S17: Equilibrium geometry of methyl ferulate *anti/cis* conformer S_2 $V'(\pi\pi^*)$ state.

Atom	X	Y	Z
C	1.399223	0.643894	0.000000
C	1.829842	1.994549	0.000000
C	0.884291	3.047687	0.000000
C	-0.496657	2.757262	0.000000
C	-0.935314	1.404393	0.000000
C	0.000000	0.332577	0.000000
C	-0.379805	-1.046667	0.000000
C	-1.653957	-1.537132	0.000000
C	-1.870454	-2.984493	0.000000
O	-3.190565	-3.280841	0.000000
C	-3.495439	-4.669410	0.000000
O	-1.004791	-3.837842	0.000000
O	1.297372	4.329103	0.000000
O	3.123135	2.409060	0.000000
C	4.151458	1.436996	0.000000
H	2.266279	4.314168	0.000000
H	5.095576	1.992923	0.000000
H	4.098705	0.803901	0.900626
H	4.098705	0.803901	-0.900626
H	2.118938	-0.173573	0.000000
H	-2.003192	1.195045	0.000000
H	0.423038	-1.790425	0.000000
H	-2.539146	-0.900992	0.000000
H	-4.589465	-4.736402	0.000000
H	-3.082546	-5.163475	-0.891989
H	-1.204683	3.584620	0.000000
H	-3.082546	-5.163475	0.891989

S_2 (ωB97XD/cc-pVDZ) DFT Energy = -726.915144894 Hartree

Table S18: Equilibrium geometry of methyl ferulate *anti/cis* conformer S_3 ($n\pi^*$) state.

Atom	X	Y	Z
C	1.336652	0.808777	0.000000
C	1.617393	2.164958	0.000000
C	0.575728	3.112313	0.000000
C	-0.739579	2.665904	0.000000
C	-1.025593	1.303377	0.000000
C	0.000000	0.341094	0.000000
C	-0.240770	-1.083813	0.000000
C	-1.493881	-1.697840	0.000000
C	-1.658341	-3.066178	0.000000
O	-2.876436	-3.624896	0.000000
C	-2.983945	-5.050127	0.000000
O	-0.663154	-3.924141	0.000000
O	0.858492	4.438340	0.000000
O	2.865207	2.728516	0.000000
C	3.978835	1.865257	0.000000
H	1.823229	4.511510	0.000000
H	4.867632	2.507384	0.000000
H	3.992425	1.225433	0.899006
H	3.992425	1.225433	-0.899006
H	2.146993	0.078936	0.000000
H	-2.070418	0.991323	0.000000
H	0.649091	-1.716548	0.000000
H	-2.428647	-1.135486	0.000000
H	-4.059218	-5.255025	0.000000
H	-2.523248	-5.483030	-0.900230
H	-1.538876	3.408213	0.000000
H	-2.523248	-5.483030	0.900230

S_3 (ωB97XD/cc-pVDZ) DFT Energy = -726.929181301 Hartree

Table S19: Equilibrium geometry of methyl ferulate *anti/trans* conformer S_0 state.

Atom	X	Y	Z
C	1.367170	0.116904	0.000000
C	2.340128	1.103986	0.000000
C	1.970388	2.463033	0.000000
C	0.622644	2.804210	0.000000
C	-0.352355	1.810971	0.000000
C	0.000000	0.457017	0.000000
C	-0.983256	-0.629843	0.000000
C	-2.320921	-0.515249	0.000000
C	-3.242095	-1.668077	0.000000
O	-2.606620	-2.858706	0.000000
C	-3.460687	-3.997660	0.000000
O	-4.447858	-1.566922	0.000000
O	2.919863	3.421987	0.000000
O	3.691780	0.907198	0.000000
C	4.166428	-0.421774	0.000000
H	3.774806	2.967555	0.000000
H	5.260651	-0.357830	0.000000
H	3.833425	-0.966387	0.899775
H	3.833425	-0.966387	-0.899775
H	1.649209	-0.936576	0.000000
H	-1.402342	2.104880	0.000000
H	-0.569956	-1.642832	0.000000
H	-2.833897	0.447657	0.000000
H	-2.796128	-4.868953	0.000000
H	-4.102784	-4.005867	0.892612
H	0.354380	3.861044	0.000000
H	-4.102784	-4.005867	-0.892612

S_0 (ωB97XD/cc-pVDZ) DFT Energy = -727.086677458 Hartree

Table S20: Equilibrium geometry of methyl ferulate *anti/trans* conformer S_1 $V(\pi\pi^*)$ state.

Atom	X	Y	Z
C	1.427467	0.303470	0.000000
C	2.135201	1.484238	0.000000
C	1.461987	2.757062	0.000000
C	0.065713	2.794651	0.000000
C	-0.659226	1.621947	0.000000
C	-0.000000	0.319840	0.000000
C	-0.740364	-0.886062	0.000000
C	-2.141804	-0.935735	0.000000
C	-2.914602	-2.157824	0.000000
O	-2.125898	-3.275375	0.000000
C	-2.836889	-4.501486	0.000000
O	-4.134485	-2.227042	0.000000
O	2.182761	3.886214	0.000000
O	3.478746	1.618152	0.000000
C	4.272599	0.442478	0.000000
H	3.118868	3.629836	0.000000
H	5.314677	0.779362	0.000000
H	4.075455	-0.160393	0.900482
H	4.075455	-0.160393	-0.900482
H	1.938371	-0.658753	0.000000
H	-1.747258	1.664903	0.000000
H	-0.193271	-1.830080	0.000000
H	-2.752463	-0.032171	0.000000
H	-2.076764	-5.292471	0.000000
H	-3.477874	-4.589233	0.890737
H	-0.426816	3.767573	0.000000
H	-3.477874	-4.589233	-0.890737

S_1 (ωB97XD/cc-pVDZ) DFT Energy = -726.935271465 Hartree

Table S21: Equilibrium geometry of methyl ferulate *anti/trans* conformer S_2 $V'(\pi\pi^*)$ state.

Atom	X	Y	Z
C	1.384667	0.050039	0.000000
C	2.387879	1.051378	0.000000
C	2.031103	2.421176	0.000000
C	0.671377	2.800734	0.000000
C	-0.339890	1.800473	0.000000
C	0.000000	0.418572	0.000000
C	-0.972557	-0.633444	0.000000
C	-2.327964	-0.469014	0.000000
C	-3.284199	-1.577371	0.000000
O	-2.687861	-2.794525	0.000000
C	-3.581906	-3.899765	0.000000
O	-4.490339	-1.441993	0.000000
O	2.986173	3.368708	0.000000
O	3.728121	0.826054	0.000000
C	4.193931	-0.509275	0.000000
H	3.840392	2.911029	0.000000
H	5.288055	-0.450408	0.000000
H	3.856271	-1.047703	0.900658
H	3.856271	-1.047703	-0.900658
H	1.648387	-1.006646	0.000000
H	-1.384391	2.105134	0.000000
H	-0.591508	-1.657774	0.000000
H	-2.799781	0.513724	0.000000
H	-2.950524	-4.795839	0.000000
H	-4.225317	-3.885919	0.892032
H	0.424068	3.861040	0.000000
H	-4.225317	-3.885919	-0.892032

S_2 (ωB97XD/cc-pVDZ) DFT Energy = -726.913136496 Hartree

Table S22: Equilibrium geometry of methyl ferulate *anti/trans* conformer S_3 ($n\pi^*$) state.

Atom	X	Y	Z
C	1.403038	0.216824	0.000000
C	2.273170	1.293626	0.000000
C	1.781350	2.613414	0.000000
C	0.408133	2.818560	0.000000
C	-0.470364	1.737854	0.000000
C	-0.000000	0.412556	0.000000
C	-0.861220	-0.748570	0.000000
C	-2.250265	-0.723627	0.000000
C	-3.028406	-1.868921	0.000000
O	-2.477605	-3.094751	0.000000
C	-3.327623	-4.244075	0.000000
O	-4.340202	-1.876837	0.000000
O	2.641197	3.661879	0.000000
O	3.640676	1.223392	0.000000
C	4.235400	-0.053816	0.000000
H	3.531686	3.283466	0.000000
H	5.319590	0.109968	0.000000
H	3.954413	-0.628939	0.898951
H	3.954413	-0.628939	-0.898951
H	1.788878	-0.803174	0.000000
H	-1.541651	1.940685	0.000000
H	-0.367667	-1.722302	0.000000
H	-2.803956	0.215253	0.000000
H	-2.646370	-5.100911	0.000000
H	-3.959121	-4.270923	0.900094
H	0.037101	3.844327	0.000000
H	-3.959121	-4.270923	-0.900094

S_3 (ωB97XD/cc-pVDZ) DFT Energy = -726.924722525 Hartree

Table S23: Equilibrium geometry of methyl ferulate *syn/cis* conformer twisted T_1 state.

Atom	X	Y	Z
C	-0.696100	0.142639	-0.328059
C	-1.975156	0.587694	-0.074173
C	-3.015619	-0.327045	0.220817
C	-2.737534	-1.691863	0.256707
C	-1.453864	-2.150627	0.004100
C	-0.396219	-1.253106	-0.298051
C	0.905376	-1.731253	-0.554306
C	2.038683	-0.883581	-0.914001
C	2.909630	-0.296733	0.097851
O	3.903696	0.427118	-0.468974
C	4.802830	1.033483	0.452096
O	2.777588	-0.421179	1.298524
O	-4.262734	0.127978	0.467397
O	-2.389592	1.888495	-0.067862
C	-1.423463	2.888611	-0.309726
H	-4.223141	1.093164	0.399220
H	-1.950018	3.847758	-0.244331
H	-0.620322	2.858747	0.445712
H	-0.981401	2.782826	-1.314885
H	0.105297	0.848136	-0.547498
H	-1.246576	-3.221683	0.037437
H	1.081742	-2.810810	-0.488212
H	2.288846	-0.682271	-1.962143
H	5.536382	1.573764	-0.156865
H	4.272595	1.728790	1.119710
H	-3.550256	-2.380808	0.489664
H	5.303814	0.273774	1.069978

T_1 (ωB97XD/cc-pVDZ) DFT Energy = -727.004202516 Hartree

Table S24: Equilibrium geometry of methyl ferulate *syn/cis* conformer T_2 state.

Atom	X	Y	Z
C	-0.993107	0.453211	0.355603
C	-2.462517	0.465870	0.181602
C	-3.226645	-0.732951	0.054307
C	-2.570866	-1.954470	0.002202
C	-1.150511	-1.940372	-0.062453
C	-0.351336	-0.799373	0.046833
C	1.077779	-0.936309	-0.024005
C	1.968673	0.091339	0.006912
C	3.406657	-0.182283	-0.024862
O	4.123492	0.966734	-0.004332
C	5.534049	0.794678	-0.031842
O	3.929918	-1.277370	-0.064577
O	-4.550937	-0.654241	-0.129467
O	-3.166578	1.583701	-0.050518
C	-2.472792	2.828018	-0.167061
H	-4.798415	0.281120	-0.081061
H	-3.245267	3.576338	-0.374109
H	-1.957258	3.070929	0.772712
H	-1.747418	2.789222	-0.989807
H	-0.543938	1.110112	1.111874
H	-0.642519	-2.900365	-0.198925
H	1.490414	-1.948259	-0.087925
H	1.650813	1.134913	0.024763
H	5.960465	1.804404	-0.011105
H	5.878639	0.217430	0.839008
H	-3.138801	-2.876665	-0.094164
H	5.849814	0.266087	-0.943523

T_2 (ωB97XD/cc-pVDZ) DFT Energy = -726.957778798 Hartree

Table S25: Equilibrium geometry of methyl ferulate *syn/cis* conformer T_4 state.

Atom	X	Y	Z
C	1.102771	0.032878	0.000000
C	2.408613	0.595170	0.000000
C	2.596123	1.997984	0.000000
C	1.489573	2.868276	0.000000
C	0.161960	2.313899	0.000000
C	-0.033360	0.906298	0.000000
C	-1.373656	0.386142	0.000000
C	-1.744609	-0.919324	0.000000
C	-3.170023	-1.273419	0.000000
O	-3.333282	-2.615110	0.000000
C	-4.685797	-3.057096	0.000000
O	-4.101088	-0.496093	0.000000
O	3.840156	2.506029	0.000000
O	3.568896	-0.110619	0.000000
C	3.505629	-1.524085	0.000000
H	4.452581	1.754719	0.000000
H	4.543720	-1.874891	0.000000
H	2.992403	-1.898455	0.900491
H	2.992403	-1.898455	-0.900491
H	0.964840	-1.044856	0.000000
H	-0.702971	2.976015	0.000000
H	-2.186227	1.119405	0.000000
H	-1.034463	-1.746637	0.000000
H	-4.643258	-4.152212	0.000000
H	-5.217343	-2.694807	0.892149
H	1.664769	3.942758	0.000000
H	-5.217343	-2.694807	-0.892149

T_4 (ωB97XD/cc-pVDZ) DFT Energy = -726.935825010 Hartree

Table S26: Equilibrium geometry of methyl ferulate *syn/cis* conformer T_5 state.

Atom	X	Y	Z
C	-0.980553	0.369422	-0.097707
C	-2.358287	0.468613	-0.009901
C	-3.158437	-0.684170	0.132431
C	-2.544908	-1.927045	0.181697
C	-1.157329	-2.028005	0.093821
C	-0.344914	-0.892779	-0.044107
C	1.096164	-1.051812	-0.125858
C	2.025364	-0.032559	-0.193808
C	3.395079	-0.211272	-0.295152
O	4.294957	0.805404	-0.372874
C	4.943947	1.140055	0.861518
O	4.031161	-1.354790	-0.361744
O	-4.505325	-0.570088	0.215245
O	-3.084021	1.627739	-0.051085
C	-2.384834	2.841212	-0.208037
H	-4.705034	0.374779	0.154759
H	-3.141746	3.634213	-0.221658
H	-1.689886	3.016525	0.631105
H	-1.820330	2.860549	-1.155954
H	-0.382724	1.271995	-0.216649
H	-0.691740	-3.014661	0.135262
H	1.453557	-2.083994	-0.125777
H	1.727449	1.018660	-0.182314
H	5.559077	2.021690	0.646034
H	4.193795	1.379402	1.631096
H	-3.170731	-2.813728	0.289181
H	5.586384	0.318500	1.209188

T_5 (ωB97XD/cc-pVDZ) DFT Energy = -726.943539601 Hartree

Table S27: Geometry of methyl ferulate E-isomer at T_1/S_0 MESX.

Atom	X	Y	Z
C	-0.80211	0.23059	-0.27472
C	-2.11598	0.55970	-0.02239
C	-3.08583	-0.44881	0.20042
C	-2.70176	-1.78831	0.16696
C	-1.38243	-2.13136	-0.08212
C	-0.39266	-1.13828	-0.31384
C	0.94536	-1.49644	-0.56472
C	2.00552	-0.54014	-0.87983
C	3.07745	-0.25589	0.06495
O	3.96637	0.62278	-0.44789
C	5.05701	0.94473	0.40905
O	3.18649	-0.74532	1.17135
O	-4.36963	-0.10699	0.44049
O	-2.63930	1.82016	0.04215
C	-1.75212	2.90660	-0.14808
H	-4.40561	0.86065	0.42404
H	-2.35693	3.81551	-0.05030
H	-0.95571	2.91274	0.61499
H	-1.29425	2.87867	-1.15131
H	0.05476	1.00629	-0.44339
H	-1.09156	-3.18309	-0.10203
H	1.20163	-2.56123	-0.59404
H	2.09401	-0.09871	-1.87967
H	5.67480	1.65037	-0.14507
H	4.70127	1.39263	1.34873
H	-3.46228	-2.55007	0.34238
H	5.64009	0.04437	0.65272

E-isomer (ωB97XD/cc-pVDZ) DFT Energy = 727.00377200 Hartree

Table S28: Geometry of methyl ferulate Z-isomer at T_1/S_0 MESX.

Atom	X	Y	Z
C	-0.80211	0.23059	-0.27472
C	-2.11598	0.55970	-0.02239
C	-3.08583	-0.44881	0.20042
C	-2.70176	-1.78831	0.16696
C	-1.38243	-2.13136	-0.08212
C	-0.39266	-1.13828	-0.31384
C	0.94536	-1.49644	-0.56472
C	2.00552	-0.54014	-0.87983
C	3.07745	-0.25589	0.06495
O	3.96637	0.62278	-0.44789
C	5.05701	0.94473	0.40905
O	3.18649	-0.74532	1.17135
O	-4.36963	-0.10699	0.44049
O	-2.63930	1.82016	0.04215
C	-1.75212	2.90660	-0.14808
H	-4.40561	0.86065	0.42404
H	-2.35693	3.81551	-0.05030
H	-0.95571	2.91274	0.61499
H	-1.29425	2.87867	-1.15131
H	0.05476	1.00629	-0.44339
H	-1.09156	-3.18309	-0.10203
H	1.20163	-2.56123	-0.59404
H	2.09401	-0.09871	-1.87967
H	5.67480	1.65037	-0.14507
H	4.70127	1.39263	1.34873
H	-3.46228	-2.55007	0.34238
H	5.64009	0.04437	0.65272

Z-isomer (ωB97XD/cc-pVDZ) DFT Energy = -727.00334800 Hartree

Table S29: Equilibrium geometry of methyl ferulate *syn/cis* conformer water cluster with *carb* orientation of the water molecule.

Atom	X	Y	Z
C	-3.164708	0.473318	0.023090
C	-1.699921	0.602241	0.021401
C	-0.918542	-0.492691	0.012993
C	0.540710	-0.547359	0.016646
C	1.154288	-1.802654	0.061454
C	2.543317	-1.925768	0.071793
C	3.337421	-0.788718	0.035512
C	2.728404	0.486300	-0.010680
C	1.351428	0.606666	-0.019674
O	-3.810044	-0.560600	0.021742
O	-3.755058	1.679204	0.027093
C	-5.180484	1.666423	0.037099
H	-1.308869	1.620846	0.034293
H	-1.414212	-1.470086	0.002626
H	0.529150	-2.697414	0.094066
H	2.520222	3.055367	0.807151
O	4.682233	-0.887358	0.043119
O	3.629695	1.511677	-0.043935
H	0.888617	1.592114	-0.057527
H	-5.486586	2.718204	0.046869
H	-5.572492	1.160970	-0.857034
H	-5.559536	1.147397	0.928925
H	5.028127	0.016743	0.014782
H	4.003110	3.491395	-0.103363
C	3.128964	2.830463	-0.084959
H	2.523803	3.000908	-0.991484
H	3.028232	-2.901483	0.108889
O	-2.755873	-3.209781	-0.060859
H	-3.247396	-2.386846	0.109682
H	-2.785063	-3.262572	-1.023202

S_0 (ωB97XD/cc-pVDZ) DFT Energy = -803.507001433 Hartree

Table S30: Equilibrium geometry of methyl ferulate *syn/trans* conformer water cluster with *carb* orientation of the water molecule.

Atom	X	Y	Z
C	3.216249	-0.007534	0.028753
C	1.750198	0.081699	-0.004858
C	0.932176	-0.984289	0.006046
C	-0.530266	-0.945441	-0.007690
C	-1.247254	-2.141941	-0.079101
C	-2.642294	-2.148003	-0.101161
C	-3.338712	-0.949620	-0.046963
C	-2.624414	0.267944	0.031473
C	-1.242686	0.271430	0.050311
O	3.949138	0.967978	0.039089
O	3.694197	-1.259525	0.049324
C	5.114386	-1.376266	0.092154
H	1.388112	1.111595	-0.033334
H	1.383059	-1.980954	0.024983
H	-0.707250	-3.089945	-0.120795
H	-2.177852	2.838794	-0.688223
O	-4.687018	-0.934316	-0.063960
O	-3.432813	1.365060	0.085629
H	-0.699209	1.213074	0.118829
H	5.322484	-2.451545	0.109933
H	5.570723	-0.911620	-0.793420
H	5.518107	-0.891718	0.992378
H	-4.954683	-0.004836	-0.013181
H	-3.631398	3.367142	0.221695
C	-2.817678	2.633972	0.186253
H	-2.212127	2.713045	1.104327
H	-3.206897	-3.078872	-0.158226
O	2.202909	3.243188	-0.183410
H	2.248004	3.262447	-1.146335
H	2.929393	2.632612	0.036698

S_0 (ωB97XD/cc-pVDZ) DFT Energy = -803.505977954 Hartree

Table S31: Equilibrium geometry of methyl ferulate *anti/cis* conformer water cluster with *carb* orientation of the water molecule.

Atom	X	Y	Z
C	-3.353176	-0.218059	-0.030090
C	-2.024439	-0.849345	-0.015698
C	-0.920546	-0.082966	0.025175
C	0.475868	-0.510957	0.032665
C	0.882996	-1.848293	0.081698
C	2.234415	-2.181244	0.080273
C	3.200411	-1.181852	0.027142
C	2.802562	0.169312	-0.022815
C	1.457628	0.499609	-0.017225
O	-3.594244	0.977320	-0.021308
O	-4.327593	-1.139629	-0.057770
C	-5.658419	-0.629409	-0.083185
H	-2.003072	-1.939544	-0.049443
H	-1.065923	1.002173	0.053549
H	0.141076	-2.646556	0.126385
H	2.991873	2.756977	0.747925
O	4.511995	-1.497376	0.023982
O	3.849249	1.044128	-0.073095
H	1.132717	1.540601	-0.058057
H	-6.312290	-1.507892	-0.108652
H	-5.861833	-0.025354	0.812552
H	-5.819437	-0.004503	-0.973149
H	4.998086	-0.660743	-0.019028
H	4.513420	2.944653	-0.184126
C	3.550224	2.423310	-0.142738
H	2.965572	2.660768	-1.047093
H	2.563644	-3.219937	0.120347
O	-1.685368	3.104353	0.124429
H	-1.739219	3.144370	1.086460
H	-2.422069	2.503988	-0.088790

S_0 (ωB97XD/cc-pVDZ) DFT Energy = -803.507503238 Hartree

Table S32: Equilibrium geometry of methyl ferulate *anti/trans* conformer water cluster with *carb* orientation of the water molecule.

Atom	X	Y	Z
C	3.304591	0.319735	-0.026681
C	1.967743	-0.292683	-0.023369
C	0.828245	0.419045	-0.033841
C	-0.533670	-0.118217	-0.040811
C	-0.811272	-1.488835	-0.100618
C	-2.123812	-1.950782	-0.101588
C	-3.183542	-1.052344	-0.043079
C	-2.918105	0.330000	0.014375
C	-1.610508	0.788692	0.014023
O	4.341577	-0.321993	-0.025260
O	3.298163	1.661180	-0.031366
C	4.581473	2.281780	-0.041848
H	1.995528	-1.384757	-0.017537
H	0.910243	1.510320	-0.034633
H	0.003044	-2.212331	-0.153840
H	-3.364261	2.878666	-0.776565
O	-4.457832	-1.495350	-0.043738
O	-4.045727	1.099450	0.065703
H	-1.399809	1.857883	0.057808
H	4.388807	3.360113	-0.050648
H	5.157376	2.002072	0.851736
H	5.150261	1.985703	-0.934597
H	-5.025448	-0.712010	-0.001683
H	-4.898540	2.923471	0.151328
C	-3.887848	2.500632	0.117916
H	-3.334458	2.808445	1.021362
H	-2.348178	-3.016645	-0.150441
O	3.553106	-3.077162	0.111577
H	3.558867	-3.116144	1.074792
H	4.012491	-2.235919	-0.059284

S_0 (ωB97XD/cc-pVDZ) DFT Energy = -803.503949980 Hartree

Table S33: Equilibrium geometry of methyl ferulate *syn/cis* conformer water cluster with *phen* orientation of the water molecule.

Atom	X	Y	Z
C	-3.892878	-0.056380	0.073387
C	-2.431685	0.145387	0.024936
C	-1.612859	-0.918509	0.002400
C	-0.151756	-0.925210	-0.045880
C	0.528444	-2.146349	-0.059724
C	1.916979	-2.190122	-0.101303
C	2.678531	-1.021612	-0.134647
C	1.994048	0.219148	-0.130026
C	0.609663	0.257095	-0.081249
O	-4.465965	-1.122549	0.098187
O	-4.538473	1.127438	0.087392
C	-5.959230	1.040513	0.132996
H	-2.079790	1.178098	0.009314
H	-2.097762	-1.900511	0.022983
H	-0.038979	-3.078803	-0.037120
H	1.502929	2.925532	0.308326
O	4.011339	-1.143759	-0.198109
O	2.782410	1.345402	-0.181234
H	0.098821	1.218900	-0.075882
H	-6.322134	2.074410	0.139077
H	-6.346815	0.503403	-0.744813
H	-6.290210	0.512725	1.039177
H	4.487005	-0.302441	-0.022780
H	2.971020	3.298351	-0.649820
C	2.159902	2.574320	-0.504961
H	1.576584	2.487653	-1.435954
H	2.453040	-3.139685	-0.114224
O	5.353533	1.049791	0.720032
H	4.492669	1.492339	0.643487
H	5.415911	0.809756	1.652503

S_0 (ωB97XD/cc-pVDZ) DFT Energy = -803.505702545 Hartree

Table S34: Equilibrium geometry of methyl ferulate *syn/trans* conformer water cluster with *phen* orientation of the water molecule.

Atom	X	Y	Z
C	3.861950	0.669024	-0.081640
C	2.389788	0.592483	-0.033617
C	1.699764	-0.559115	0.006840
C	0.245961	-0.720780	0.056174
C	-0.299874	-2.007072	0.085917
C	-1.675884	-2.198909	0.128595
C	-2.558340	-1.118573	0.147736
C	-2.010275	0.188189	0.127294
C	-0.637803	0.373350	0.077214
O	4.480872	1.708735	-0.119187
O	4.458922	-0.540274	-0.080016
C	5.881546	-0.511701	-0.125658
H	1.909925	1.572617	-0.034683
H	2.275902	-1.488925	0.002467
H	0.363318	-2.874403	0.074746
H	-1.808305	2.921843	-0.355507
O	-3.870336	-1.382460	0.213594
O	-2.913706	1.224527	0.165502
H	-0.233251	1.384190	0.059688
H	6.201133	-1.559761	-0.117784
H	6.234127	-0.010570	-1.038709
H	6.290726	0.020069	0.745474
H	-4.434081	-0.599635	0.027954
H	-3.307589	3.154980	0.598715
C	-2.424354	2.518465	0.465470
H	-1.835161	2.511119	1.396679
H	-2.107075	-3.200219	0.153701
O	-5.439920	0.644658	-0.721837
H	-4.632193	1.178767	-0.649764
H	-5.479008	0.396270	-1.653375

S_0 (ωB97XD/cc-pVDZ) DFT Energy = -803.504374127 Hartree

Table S35: Equilibrium geometry of methyl ferulate *anti/cis* conformer water cluster with *phen* orientation of the water molecule.

Atom	X	Y	Z
C	-4.004585	0.266310	0.076879
C	-2.710546	-0.441035	0.008100
C	-1.570150	0.266875	0.000322
C	-0.200592	-0.242394	-0.063307
C	0.114882	-1.604661	-0.135996
C	1.436410	-2.020987	-0.189205
C	2.495657	-1.107101	-0.177390
C	2.183298	0.268950	-0.114324
C	0.858027	0.680740	-0.052647
O	-4.159164	1.466425	0.130373
O	-5.029264	-0.608651	0.073082
C	-6.325888	-0.023285	0.136368
H	-2.744062	-1.530514	-0.034272
H	-1.682462	1.355829	0.047825
H	-0.677613	-2.353754	-0.151459
H	2.444858	2.996820	0.411570
O	3.740556	-1.597522	-0.254974
O	3.248629	1.140678	-0.121242
H	0.619785	1.743452	0.000413
H	-7.032681	-0.860458	0.124166
H	-6.444989	0.565478	1.057499
H	-6.499513	0.637432	-0.725376
H	4.432138	-0.932850	-0.042198
H	3.966170	2.980296	-0.535268
C	2.987246	2.501290	-0.410886
H	2.409824	2.601886	-1.344085
H	1.692064	-3.079646	-0.247557
O	5.625075	0.100774	0.749539
H	5.604434	-0.174369	1.674129
H	4.922839	0.768553	0.682903

S_0 (ωB97XD/cc-pVDZ) DFT Energy = -803.505497065 Hartree

Table S36: Equilibrium geometry of methyl ferulate *anti/trans* conformer water cluster with *phen* orientation of the water molecule.

Atom	X	Y	Z
C	-4.116778	-0.369492	0.070304
C	-2.722700	-0.849074	0.014503
C	-1.654472	-0.035657	-0.005617
C	-0.244731	-0.428282	-0.060254
C	0.186247	-1.759490	-0.109926
C	1.538621	-2.062542	-0.155745
C	2.516287	-1.062026	-0.158827
C	2.087495	0.282994	-0.118197
C	0.731717	0.581394	-0.064353
O	-5.081011	-1.100396	0.087981
O	-4.217106	0.976234	0.100462
C	-5.547371	1.480088	0.154505
H	-2.639972	-1.936691	-0.009122
H	-1.847109	1.041047	0.022113
H	-0.538445	-2.574361	-0.113220
H	2.115390	3.034278	0.353985
O	3.798688	-1.445346	-0.228044
O	3.074975	1.242034	-0.137516
H	0.405027	1.621185	-0.029033
H	-5.452408	2.571569	0.176595
H	-6.122458	1.163793	-0.727762
H	-6.065612	1.122458	1.055966
H	4.430864	-0.719425	-0.031562
H	3.637114	3.126658	-0.587399
C	2.701558	2.569171	-0.456243
H	2.122271	2.601618	-1.393197
H	1.882893	-3.096606	-0.196606
O	5.535991	0.424598	0.737456
H	4.776661	1.026483	0.670792
H	5.548249	0.158494	1.664836

S_0 (ωB97XD/cc-pVDZ) DFT Energy = -803.503624641 Hartree

Table S37: Equilibrium geometry of methyl ferulate *syn/cis* conformer water cluster with *phen2* orientation of the water molecule.

Atom	X	Y	Z
C	-0.844381	0.566640	0.024748
C	-2.147077	0.088882	0.026144
C	-2.377200	-1.302130	0.011534
C	-1.294102	-2.173879	-0.004648
C	0.011730	-1.687804	-0.005743
C	0.255823	-0.313002	0.009237
C	1.603632	0.261564	0.009456
C	2.773580	-0.394445	-0.003671
C	4.037761	0.370553	-0.000729
O	5.098129	-0.459575	-0.016486
C	6.370099	0.182024	-0.015574
O	4.139209	1.576819	0.013696
O	-3.673450	-1.699352	0.012899
O	-3.266619	0.849618	0.040719
C	-3.144717	2.257742	-0.014841
H	-3.707082	-2.661508	-0.029688
H	-4.178220	2.620843	-0.047058
H	-2.628607	2.650671	0.877416
H	-2.605825	2.577713	-0.922397
H	-0.658852	1.640993	0.035756
H	0.839429	-2.397110	-0.018881
H	1.665918	1.355226	0.021841
H	2.855442	-1.482178	-0.016912
H	7.111451	-0.624558	-0.029970
H	6.484484	0.824072	-0.900871
H	-1.475344	-3.251854	-0.017171
H	6.494629	0.801505	0.884338
O	-6.077979	0.920187	-0.127186
H	-6.181621	0.853667	0.828573
H	-5.240992	0.454086	-0.269596

S_0 (ωB97XD/cc-pVDZ) DFT Energy = -803.494118049 Hartree

Table S38: Equilibrium geometry of methyl ferulate *anti/cis* conformer water cluster with *phen2* orientation of the water molecule.

Atom	X	Y	Z
C	0.599590	-0.144088	0.016728
C	1.979116	-0.029364	0.023119
C	2.579069	1.251465	0.012107
C	1.774984	2.381483	-0.005579
C	0.383837	2.258399	-0.011473
C	-0.221804	1.002586	-0.000066
C	-1.683359	0.920194	-0.006224
C	-2.442597	-0.185847	0.005345
C	-3.914718	-0.061504	-0.003714
O	-4.496299	-1.276476	0.010184
C	-5.920873	-1.266071	0.002504
O	-4.541416	0.973998	-0.021013
O	3.934069	1.277935	0.018107
O	2.856095	-1.059767	0.039231
C	2.365383	-2.384957	-0.013678
H	4.230218	2.194255	-0.021262
H	3.265618	-3.009340	-0.039570
H	1.759294	-2.623380	0.876746
H	1.764962	-2.553596	-0.923427
H	0.143760	-1.133069	0.024447
H	-0.234826	3.157237	-0.025474
H	-2.220100	1.874807	-0.021855
H	-2.035207	-1.197786	0.021410
H	-6.227423	-2.317847	0.016703
H	-6.312545	-0.740247	0.885223
H	2.238057	3.371510	-0.015448
H	-6.303080	-0.767282	-0.899841
O	5.548067	-1.875143	-0.117308
H	5.666899	-1.832600	0.838056
H	4.868467	-1.200813	-0.263724

S_0 (ωB97XD/cc-pVDZ) DFT Energy = -803.494771886 Hartree
